# RACK1A interacts and colocalizes with FSD1 in stress granules to regulate salt stress response in Arabidopsis

**DOI:** 10.1093/plphys/kiaf659

**Published:** 2025-12-17

**Authors:** Pavol Melicher, Petr Dvořák, Maryna Tsinyk, Jan Řehák, Olga Šamajová, Kateřina Hlaváčková, Miroslav Ovečka, Jozef Šamaj, Tomáš Takáč

**Affiliations:** Department of Biotechnology, Faculty of Science, Palacký University Olomouc, Olomouc, Czech Republic; Department of Biotechnology, Faculty of Science, Palacký University Olomouc, Olomouc, Czech Republic; Department of Biotechnology, Faculty of Science, Palacký University Olomouc, Olomouc, Czech Republic; Department of Biotechnology, Faculty of Science, Palacký University Olomouc, Olomouc, Czech Republic; Department of Biotechnology, Faculty of Science, Palacký University Olomouc, Olomouc, Czech Republic; Department of Biotechnology, Faculty of Science, Palacký University Olomouc, Olomouc, Czech Republic; Department of Biotechnology, Faculty of Science, Palacký University Olomouc, Olomouc, Czech Republic; Department of Biotechnology, Faculty of Science, Palacký University Olomouc, Olomouc, Czech Republic; Department of Biotechnology, Faculty of Science, Palacký University Olomouc, Olomouc, Czech Republic

## Abstract

The generation of reactive oxygen species (ROS) and their regulation by antioxidant enzymes, such as IRON SUPEROXIDE DISMUTASE 1 (FSD1), are critical for managing plant responses to salt stress. However, the protein networks modulating ROS levels during salt stress remain incompletely understood. Our co-immunoprecipitation analysis identified FSD1 as an interaction partner of the scaffolding protein RECEPTOR FOR ACTIVATED C KINASE 1A (RACK1A). Bimolecular fluorescence complementation analysis revealed that RACK1A interacts with FSD1 predominantly in the cytoplasm. Despite elevated FSD1 activity in *rack1a* mutants, the abundance of FSD1 protein remained unchanged. Additionally, we found that the RACK1A-FSD1 module was involved in root hair tip growth, highlighting the developmental significance of this interaction. While *rack1a* mutants exhibited salt resilience, the *fsd1-1 rack1a-1* double mutant displayed reduced salt stress resistance, which was substantiated by reduced ROS levels. Upon salt stress, a distinct pool of RACK1A and FSD1 proteins accumulated in cycloheximide-sensitive structural condensates in the cytoplasm that colocalized with the stress granule (SG) marker protein RNA-BINDING PROTEIN 47. FSD1 activity was lower in SGs compared to the soluble extract. RACK1A also interacted with TUDOR STAPHYLOCOCCAL NUCLEASE 2, which participates in SG formation. However, *RACK1A* knock-out completely abolished salt-stress-dependent accumulation of FSD1 in structural condensates, suggesting that RACK1A likely mediates the recruitment of FSD1 to SGs. Thus, this study uncovers a mechanism for the regulation of RACK1/FSD1-dependent antioxidant defense in response to salt stress in *Arabidopsis thaliana*.

## Teaser

A scaffolding protein accumulates in stress granules during salt stress to recruit an antioxidant enzyme, thereby modulating its activity and improving seedling salt stress resistance.

## Introduction

Salt stress represents a severe constraint harming crop production (Hassani et al. 2021). Plants respond to salt stress by complex mechanisms governed by diverse signaling networks and second messengers, including reactive oxygen species (ROS) (Ovečka et al. 2014; Van Zelm et al. 2020; Singh 2022). To regulate ROS levels, plants evolved a complex antioxidant system. Thus, gene modifications leading to antioxidant capacity reinforcement usually result in elevated salt stress tolerance (Zhang et al. 2014; Yan et al. 2016).

Superoxide dismutases (SODs) are key enzymatic antioxidants that catalyze the dismutation of superoxide into hydrogen peroxide (H_2_O_2_) (Pilon et al. 2011; Dreyer and Schippers 2019). Recently, we demonstrated that the control of superoxide levels by *Arabidopsis thaliana* IRON SUPEROXIDE DISMUTASE 1 (FSD1) is inevitable for the efficient antioxidant defense during oxidative stress response (Dvořák et al. 2021 ; Melicher et al. 2022 , 2024). FSD1 is also involved in plant responses to salt stress, while its absence in *fsd1* mutants leads to a high accumulation of ROS and salt hypersensitivity (Dvořák et al. 2021). Nevertheless, molecular mechanisms regulating FSD1 compartmentalization and activity during salt stress are not entirely understood.

FSD1 and COPPER-ZINC SUPEROXIDE DISMUTASE 1 (CSD1) were identified by large-scale yeast 2-hybrid screens as interactors of RECEPTOR FOR ACTIVATED C KINASE 1 A (RACK1A) (Guo et al. 2011). RACK1 is an evolutionarily conserved cytosolic protein containing 7 WD40 motifs forming a 7-bladed β-propeller structure that facilitates protein–protein interactions (PPIs) (Guo et al. 2019). Arabidopsis genome contains 3 *RACK1* isoforms: *RACK1A, RACK1B,* and *RACK1C* (Chen et al. 2006). *RACK1A* is a predominant isoform, as demonstrated by the pronounced phenotypes of the *rack1a* mutant (Guo and Chen 2008). Plant RACK1 isoforms interact with proteins involved in plant development and responses to environmental cues (Islas-Flores et al. 2015; Li et al. 2023, 2024; Masood et al. 2023; Fu et al. 2024) and act as a scaffold proteins orchestrating mitogen-activated protein kinase and brassinosteroid signaling pathways during stress responses (Cheng et al. 2015; Li et al. 2023). It might be involved in 60S and 80S ribosome assembly and translation by interaction with EUKARYOTIC INITIATION FACTOR 6 (Guo et al. 2011).

RACK1 is also implicated in salt stress response. Upon salt stress, UNIVERSAL STRESS PROTEIN 17 monomerizes and relocates to the nucleus, where it interacts with RACK1C, negatively affecting the expression of abscisic acid-mediated salt stress-responsive genes in Arabidopsis (Bhuria et al. 2022). Furthermore, overexpression of *Brassica oleracea RACK1* increased plant tolerance to the salt stress (Li et al. 2017), while *RACK1A*-overexpressing rice (*Oryza sativa*) and soybean (*Glycine max*) lines were hypersensitive to the salt stress (Zhang et al. 2018; Zheng et al. 2019 ). This suggests distinct roles of RACK1 in the salt stress tolerance of different plant species.

*RACK1* expression often correlates with the expression of several genes encoding antioxidant enzymes. In soybean, RNAi-mediated downregulation of *GmRACK1* was shown to positively affect *CATALASE, SOD, ASCORBATE PEROXIDASE*, and *GLUTATHIONE S-TRANSFERASE* transcript levels and SOD, peroxidase and catalase activities under salt and polyethylenglycol-mediated drought stress (Zheng et al. 2019), while in rice, downregulation of *OsRACK1* reduced membrane peroxidation and enhanced SOD activity during drought stress (Li et al. 2009). Although the findings indicate the involvement of RACK1 in the regulation of antioxidant enzymes, the specific mechanisms of RACK1 contribution remain unclear.

In mammalian cells, RACK1 is sequestered to stress granules (SGs) to inhibit the apoptotic pathway (Arimoto et al. 2008). In plants, RACK1B isoform has been identified in the interactome of TUDOR STAPHYLOCOCCAL NUCLEASE 2 (TSN2) under non-stressed conditions, and the bimolecular fluorescence complementation (BiFC) analysis indicated the TSN2-RACK1B interaction in heat stress-induced SGs (Gutierrez-Beltran et al. 2021). SGs are membrane-less cytoplasmic condensates of proteins, RNAs, and metabolites that are formed after stress treatments (Maruri-López et al. 2021 ). They facilitate the plant stress responses by inhibiting translation, mediating mRNA turnover, and protecting proteins from unfolding or degradation (Vanderweyde et al. 2013; Protter and Parker 2016). Assembly of plant SGs has been reported during responses to various environmental stresses, including heat, hypoxia, or salinity (Weber et al. 2008; Sorenson and Bailey-Serres 2014; Gutierrez-Beltran et al. 2015). OLIGOURIDYLATE BINDING PROTEIN 1B (UBP1B), a component of SGs, interacts with 3′-UTR of target mRNA, providing protection against degradation under stress (Nguyen et al. 2017). Overexpression of *UBP1B* positively affected heat tolerance, while its mutation resulted in increased sensitivity to salt, heat, and osmotic stress (Weber et al. 2008; Nguyen et al. 2017). TSN1 is a docking platform for SG components and affects salt and heat stress responses. TSNs function in mRNA decapping, affecting the structural integrity and molecular composition of SGs. Overexpression of *TSN1* enhanced salt stress tolerance, while *TSN1* deficiency resulted in increased sensitivity to both salt and heat stress (Gutierrez-Beltran et al. 2015). ANGUSTIFOLIA (AN) was found to be recruited to SGs during salt, osmotic or heat stress conditions. Mutants impaired in *AN* expression exhibited differences in SG structure under heat stress and were more tolerant to salt and osmotic stress (Bhasin and Hülskamp 2017).

The functional implications of RACK1A-FSD1 interaction may represent a key component of the plant antioxidant activity, but it remains largely unexplored. Here, we provide evidence that RACK1A interacts with and modulates the activity of FSD1. We revealed that RACK1A negatively regulates salt stress tolerance in Arabidopsis and accumulates in cytoplasmic condensates identified as SGs. In addition, RACK1A likely mediates the recruitment of FSD1 to SGs, which likely contributes to the regulation of ROS levels during the salt stress response. Therefore, RACK1A plays a crucial role in the salt stress response in Arabidopsis, where the mechanism includes its interaction with FSD1 and accumulation in SGs.

## Results

### Microscopic analysis of tissue-specific and developmental RACK1A-GFP localization in Arabidopsis roots

Prior to examination of RACK1A-FSD1 interaction, we conducted a detailed tissue-specific and developmental localization of RACK1A-GFP in roots of *rack1a-1* mutant complemented with *proRACK1A::RACK1A:GFP* construct, to point out similarities in developmental functions with FSD1. Fluorescence signal of cellular RACK1A-GFP presence was observed in all plant organs of 5-d-old seedlings, while the strongest signal was observed in organs and tissues with high meristematic activity, such as root tip, lateral root primordia, and developing true leaves (Supplementary Fig. S1).

**Figure 1.**
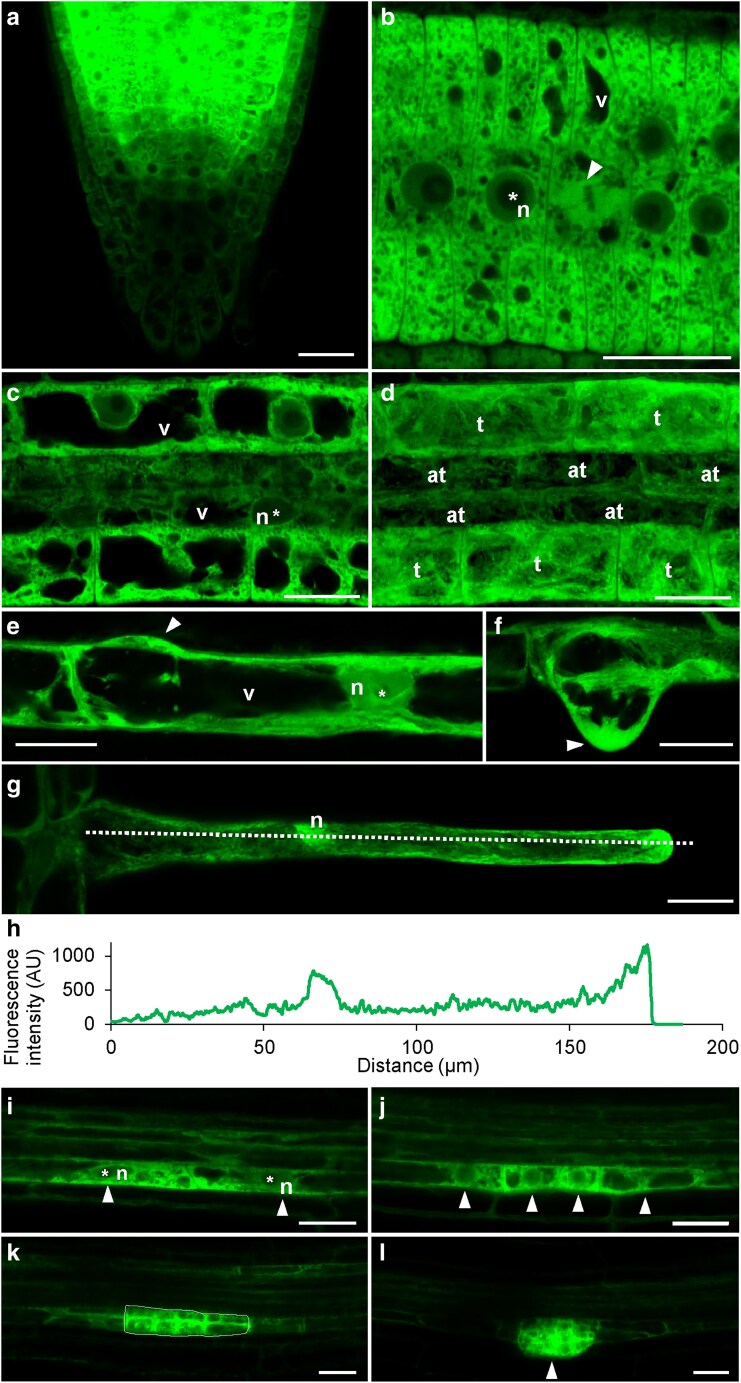
Airyscan confocal laser scanning microscopy (ACLSM) of RACK1A-GFP localization in Arabidopsis root. (a) Root tip with a high abundance of RACK1A in the meristematic zone. (b) Root meristematic cells. White arrowhead points to the dividing cell at the metaphase. (c and d) Epidermal cells of root elongation zone—single plane image (c), orthogonal projection of 20 optical planes (d). (e–h) RACK1A-GFP localization in a trichoblast at early (e) and late (f) bulging stage. Fluorescent RACK1A-GFP signal was located in a cytoplasm, particularly at the forming root hair tip (arrowheads in e, f) and in nucleus (n). Unlike that, nucleoli (*) and vacuoles (v) were devoid of RACK1A-GFP. In growing root hairs, RACK1A-GFP was located in cytoplasm, nucleus (n) and growing tip (g), as evidenced by a profile-based measurement of the fluorescence intensity distribution (h) along the line shown in (g). (i) Initiation of lateral root primordia formation. White arrowheads indicate 2 lateral root founder cells with increased RACK1A-GFP fluorescence intensity in cytosol. (j) Lateral root primordia cells after first anticlinal division. White arrowheads indicate 4 lateral root primordia cells with increased RACK1A-GFP fluorescence intensity in cytosol. (k) Initiation of endodermis crossing after periclinal division of primordia cells. White line indicates lateral root primordia cells with increased RACK1A-GFP fluorescence intensity. (l) Cortex/epidermis crossing and emergence of the lateral root. White arrow indicates the lateral root tip. n, nucleus; *, nucleolus; v, vacuole; t, trichoblast; at, atrichoblast. Scale bar = 20 μm.

In the root tip, RACK1A-GFP exhibited high abundance in meristematic zone (Fig. 1a). However, in cells of quiescent center, stem cell niche, columella, and lateral root cap, the fluorescence intensity was substantially lower (Fig. 1a). On the subcellular level, both in the root meristematic (Fig. 1b) and elongation (Fig. 1c and d) zones, a high abundance of RACK1A-GFP was observed in the cytosol, while in the nucleus, the fluorescence intensity was lower.

In the root elongation zone, the abundance of RACK1A-GFP was significantly higher in trichoblasts compared to atrichoblasts (Fig. 1d), suggesting an involvement of RACK1A in root hair formation. During root hair growth, RACK1A-GFP was accumulated in the cortical cytoplasm of the bulging site at the basal end of trichoblasts (Fig. 1e), in root hair bulges (Fig. 1f) and actively growing root hairs (Fig. 1g). At the subcellular level, RACK1A-GFP localization was abundant in nuclei and at the growing root hair tip (Fig. 1g and h). Note that a similar pattern in growing root hairs was observed also for FSD1-GFP (Dvořák et al. 2021). Dynamic mode of developmental RACK1A-GFP localization during root hair formation was revealed by live-cell light-sheet fluorescence microscopy (LSFM) root imaging (Supplementary Figs S2a and S3a–f; Supplementary Video S1). Involvement of RACK1A in different stages of root hair development was documented by a tip-focused localization of RACK1A-GFP in bulges, emerging, and actively-growing root hairs. Importantly, it disappeared from the tip in fully-grown root hairs with terminated tip growth (Supplementary Fig. S2a; Supplementary Videos S1 and S2; Supplementary Fig. S3a–f). Subcellular pattern of RACK1A-GFP fluorescence intensity distribution was confirmed by a pseudo-color-coding visualization (Supplementary Fig. S2b; Supplementary Videos S3 and S4; Supplementary Fig. S3g–l) and by a kymograph-based semi-quantitative evaluation of growing root hairs (Supplementary Fig. S2c–f), evidencing a tip-focused RACK1A-GFP accumulation.

Airyscan confocal laser scanning microscopy (ACLSM) detection revealed that RACK1A may be involved in the lateral root initiation (Fig. 1i–l). Within the pericycle, RACK1A-GFP signal appeared substantially increased in lateral root founder cells, even before the first anticlinal cell division (Fig. 1i). Subsequently, high abundance of RACK1A-GFP was retained in newly formed cells after the first anticlinal cell division (Fig. 1j), which was also apparent during all subsequent phases of lateral root primordia development (Fig. 1k and l, Supplementary Video S5) as indicated by pseudo-color-coding visualization (Supplementary Video S6). The enrichment of the fluorescent signal in lateral root primordia was also observed for FSD1-GFP (Dvořák et al. 2021).

### RACK1A interacts with FSD1, predominantly in the cytosol

Published high-throughput yeast 2-hybrid screening identified FSD1 as a potential interactor of RACK1A (Guo et al. 2019). We adopted 2 independent methods to further validate this interaction.

**Figure 2.**
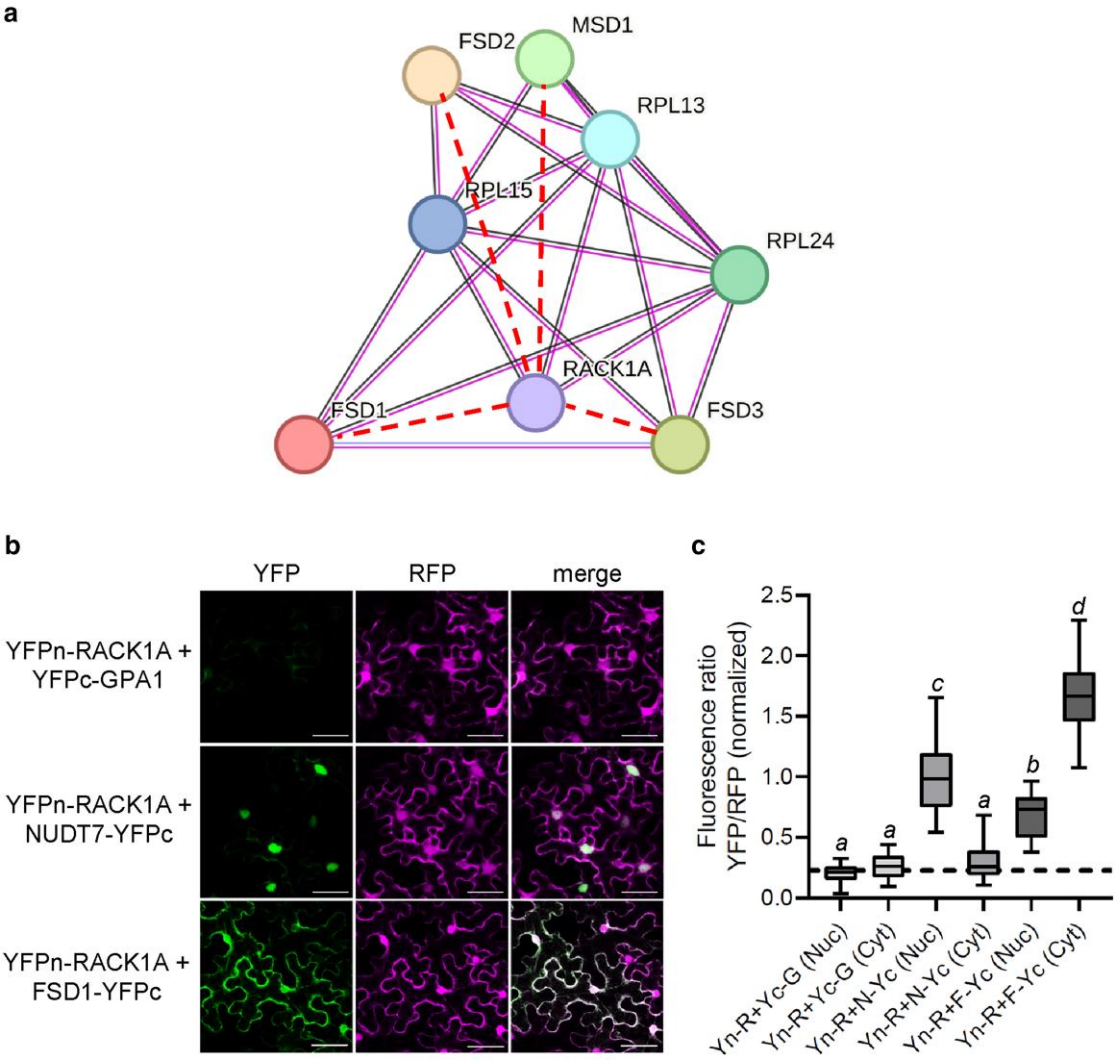
Functional validation of FSD1-RACK1A interaction. (a) Salt-induced RACK1A protein interaction network with superoxide dismutases as constructed by the STRING web-based application. The network was extracted from a protein interaction network constructed using the full RACK1A-interactome, as shown in Supplementary Fig. S5. RPL = refers to isoforms of 50S RIBOSOMAL PROTEIN L. (b and c) Protein-protein interaction (PPI) study of RACK1A with FSD1 by ratiometric bimolecular fluorescence complementation assay (rBiFC). (b) Representative images of rBiFC assay taken by confocal laser scanning microscopy (CLSM). Artificial green color for YFP indicates the PPI, while RFP signal (magenta) represents a control of transformation and reference for calculations of relative PPI strength. Scale bars = 20 µm. (c) Relative quantification of PPI strength measured as a ratio of YFP to RFP relative fluorescence intensity in nuclei (Nuc) and cytosol (Cyt). Ratio values were normalized to the mean ratio measured in nuclei of positive control transformed with vectors carrying *YFPn-RACK1A+NUDT7-YFPc* (Yn-R+N-Yc) genes. The dashed line represents the mean ratio measured in negative controls transformed with vectors carrying YFPn-RACK1A+YFPc-GPA1 (Yn-R+Yc-G) and functions as a threshold of positive interaction. Vector carrying *YFPn-RACK1A+FSD1-YFPc* (Yn-R+F-Yc) genes was used to study the interaction of RACK1A with FSD1. Centre lines of the boxes represent median values. Upper and lower box limits represent upper and lower quartiles, respectively. Whiskers represent maximum and minimum values. Italic letters indicate a statistically significant difference at a *P* < 0.05 as determined by 1-way ANOVA with post-hoc Tukey HSD test.

First, since both proteins participate in plant salt stress responses, we analyzed the salt-induced interactome of RACK1A-GFP in a stably transformed Arabidopsis line using GFP trap technology coupled with MS/MS (Supplementary Datasets S1 and S2). The salt induced interactome included proteins associated with oxidative stress responses, such as FSD1, FSD2, FSD3, MANGANESE SUPEROXIDE DISMUTASE 1 (MSD1; Fig. 2a), ASCORBATE PEROXIDASE 3, secretory peroxidases, and peroxiredoxins (Supplementary Dataset S2). The extraction of interaction networks connecting RACK1A, FSD1, FSD2, FSD3 and MSD1 indicated their coexpression with plastid 50S RIBOSOMAL PROTEIN L 13 (RPL13), RPL15, and RPL24 (Fig. 2a). These findings further validate the interaction between RACK1A and FSD1. Hence, a substantial portion of the RACK1A-GFP interactome was salt stress-dependent, as evidenced by the higher number of protein interaction clusters identified by STRING compared to the interactome of the mock-treated control plants (Supplementary Figs. S4 and S5). The salt-induced RACK1A interactome was enriched with proteins involved in ribosome biogenesis, Golgi vesicle transport, metabolism, and gene expression (Supplementary Fig. S6). Gene ontology annotations uniquely induced by salt stress included Golgi vesicle transport, amino acid metabolism, and cell tip growth (Supplementary Datasets S3 and S4). The salt stress-induced and control interactomes included also TSN1 and TSN2, serving as a docking platform for SG components (Gutierrez-Beltran et al. 2021).

Next, we validated the interaction between RACK1A and FSD1 using a ratiometric bimolecular fluorescence complementation (rBiFC) assay (Fig. 2b and c). The presence of RFP fluorescence confirmed the successful transient expression of all expression vectors containing the genes of interest in the leaf epidermal cells of *Nicotiana benthamiana* (Fig. 2b). The lack of YFP fluorescence in samples expressing the negative control (RACK1A-GPA1) and the presence of YFP fluorescence in samples with the positive control (RACK1A-NUDT7) validated the functionality of the method. The interaction between RACK1A and FSD1 was predominantly localized in the cytosol (Fig. 2b), with the ratio of YFP to RFP fluorescence intensity being higher than that observed in the positive control measurements (Fig. 2c). Notably, the abundance of nuclear YFP signal was significantly lower compared to the signal measured in the positive control, indicating that the RACK1A-FSD1 interaction primarily occurs in the cytosol and is considerably less abundant in the nucleus.

We also proved the interaction between TSN1 and RACK1A, which predominantly localized to the cytosol (Supplementary Fig. S7a), with the ratio of YFP to RFP fluorescence intensity being higher than that observed in the positive control measurements (Supplementary Fig. S7b). Similar to the RACK1A-FSD1 interaction, the nuclear YFP signal for RACK1A-TSN1 was significantly lower than that observed in the positive control.

### SOD activities and abundances in *rack1a* mutants and *fsd1-1 rack1a-1* double mutant

To study genetic interaction between RACK1A and FSD1, a double mutant lacking both *FSD1* and *RACK1A* was generated through conventional crossing, and the absence of these proteins was confirmed via immunoblotting (Supplementary Fig. S8a and b). Functional genetic experiments also included single *fsd1-1* and *rack1a-1* mutants, as well as the CRISPR/Cas9 knock-out mutant *rack1a-5*, all lacking corresponding proteins (Supplementary Fig. S8a and b).

**Figure 3.**
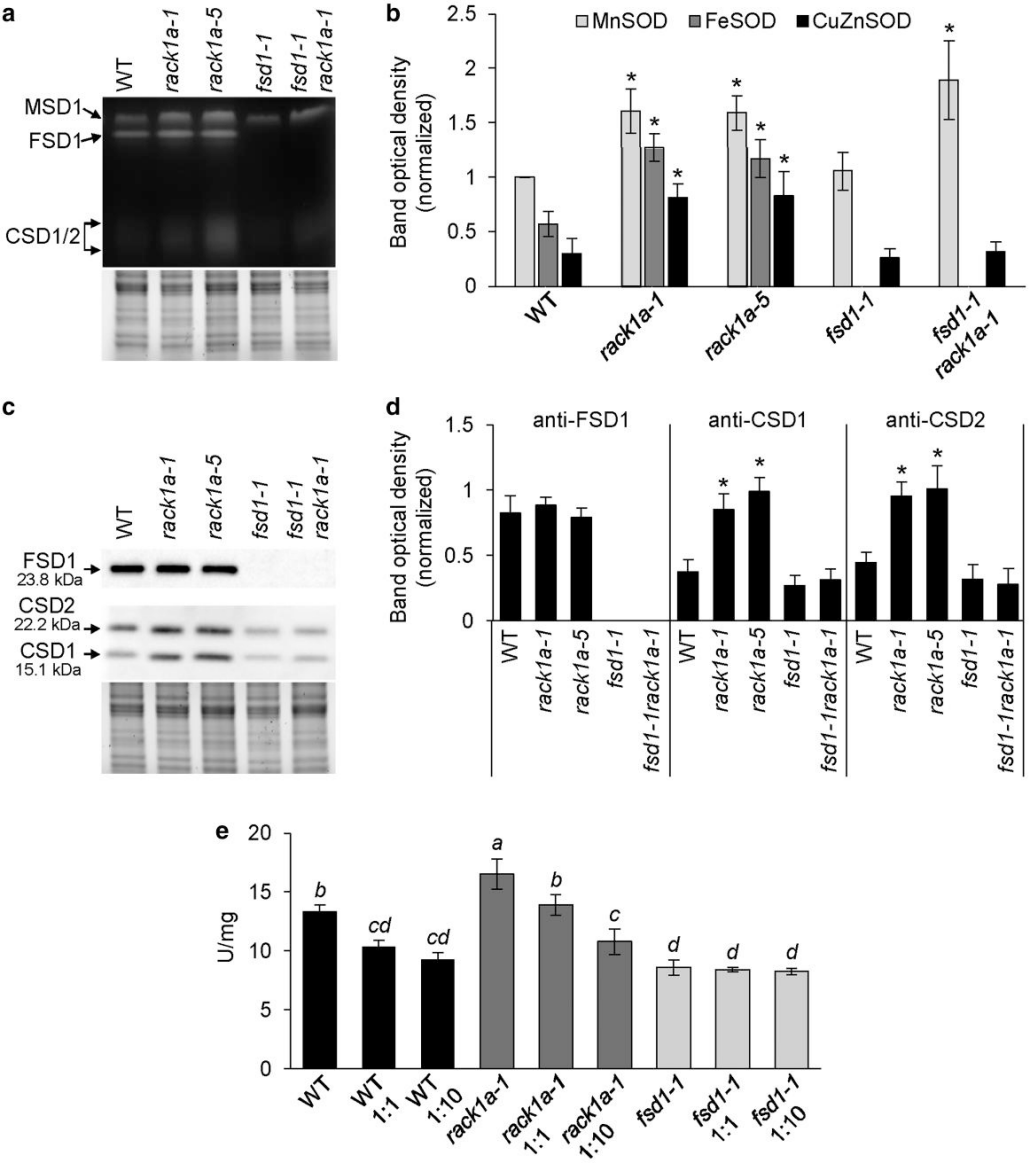
Isoenzyme activity measurement and immunoblotting of FSD1, CSD1 and CSD2 in roots of 14-d-old wild type (WT), *rack1a-1*, *rack1a-5*, *fsd1-1* mutants and *fsd1-1 rack1a-1* double mutant grown on ½ MS media. (a) In-gel SOD activity staining supplemented with respective controls of protein loading using Stain-free gel. (b) Quantification of band optical density in (a). Values in (b) are expressed as relative to the activity of MSD1 (mean ± SD, *N* = 4). (c) Immunoblots of FSD1, CSD1 and CSD2 using anti-FSD1 and CSD2 antibody, supplemented with respective controls of protein loading using Stain-free gels. (d) Quantification of band optical density in (c). Values in (d) are expressed as relative to the abundance of the protein in WT (mean ± SD, *N* = 4). Asterisks above the columns in (b) and (d) indicate a statistically significant difference between WT and respective line as revealed by one-way ANOVA with post-hoc Tukey HSD test (*P* < 0.05). (e) Total SOD activity measurement with and without the addition of recombinant RACK1A protein in 1:1 or 1:10 protein content ratio. Different letters above the columns indicate a statistically significant difference as revealed by 1-way ANOVA with post-hoc Tukey HSD test (*P* < 0.05; mean ± SD, *N* = 3).

Abundances and activities of SOD isozymes were evaluated in the roots of plants grown under limited Cu^2+^ availability (0.05 µM) (Fig. 3), causing increased *FSD1* expression, while minimizing the impact of *CSD1*, which is under such conditions weakly expressed (Melicher et al. 2022). As expected, the in-gel SOD activity assay revealed that MSD1 exerted the highest activity among the SOD isozymes in WT (Fig. 3a and b). On the other hand, the activities of SOD isozymes in both *rack1a* mutants were significantly higher compared to WT (Fig. 3a and b). The activity of MSD1 was 1.6-times higher in both *rack1a-1* and *rack1a-5* mutants, respectively. The activities of FSD1 and both CSDs were 2.2- and 2.7-times higher, respectively, in *rack1a-1* mutant compared to WT. Comparably, in *rack1a-5* mutant, FSD1 activity was 2-times higher, while CSDs activity was increased up to 2.7-times. In the *fsd1-1 rack1a-1* double mutant, the activity of MSD1 was 2-fold higher compared to the WT, whereas the *fsd1-1* mutant showed results similar to the WT (Fig. 3a and b). These data suggest the regulation of SOD isozymes activities by RACK1A.

Immunoblotting showed that in both *rack1a* mutants, the abundances of FSD1 were similar to WT (Fig. 3c and d). On the other hand, increased abundances of CSD1 and CSD2 were observed in both *rack1a-1* and *rack1a-5* mutants (Fig. 3c and d). Thus, FSD1 activity may be regulated by RACK1A, possibly through their interaction.

To further test such hypothesis, we prepared recombinant GST-RACK1A (Supplementary Fig. S9) and measured total SOD activity in root extracts isolated from WT, *rack1a-1*, and *fsd1-1* lines *in vitro* in the presence of this recombinant protein. The results showed elevated SOD activity in the *rack1a-1* mutant compared to the WT, whereas the *fsd1-1* mutant contained the lowest SOD activity (Fig. 3e). Adding recombinant GST-RACK1A (at a 1:1 protein ratio) significantly reduced total SOD activity in WT and *rack1a-1* extracts, but had no effect on *fsd1-1*. A 10-times higher dose of GST-RACK1A further decreased SOD activity in extracts from *rack1a-1* (Fig. 3e). These findings confirm that RACK1A negatively regulates FSD1, as the *fsd1-1* mutant did not show any reduction in total SOD activity upon addition of recombinant RACK1A, unlike WT and *rack1a-1*.

### Both single *rack1a* mutants and the *fsd1-1 rack1a-1* double mutant have altered phenotypes

**Figure 4.**
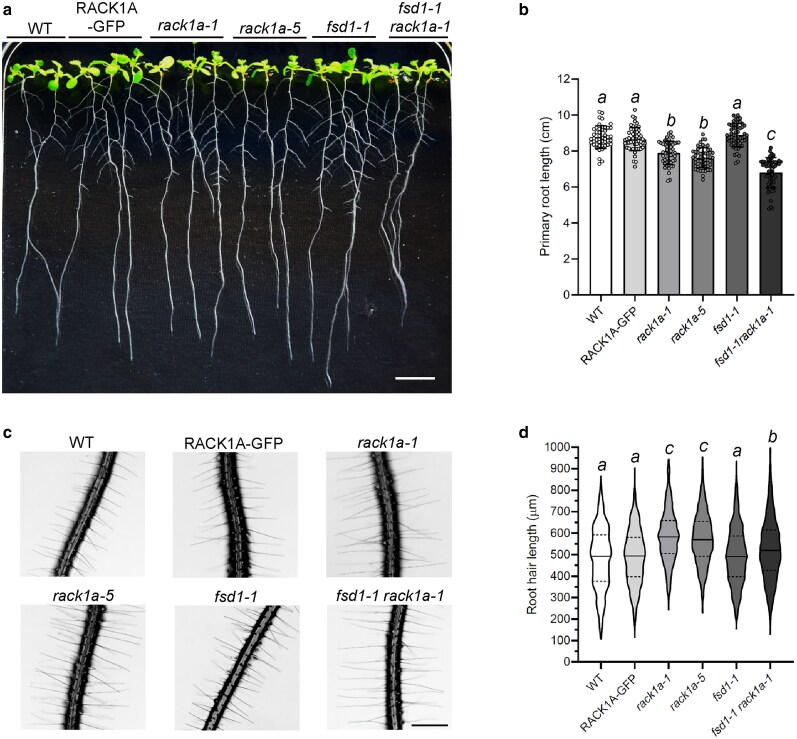
Phenotypic analysis of Col-0 (wild type; WT), RACK1A-GFP-complemented *rack1a-1* mutant (RAK1A-GFP), *rack1a-1*, *rack1a-5, fsd1-1* mutants, and *fsd1-1 rack1a-1* double mutant. (a) Representative picture of 10-d-old seedlings. (b) Quantification of primary root length of 10-d-old seedlings (*N* = 50). Circle symbols represent individual values. Error bars represent standard deviation. Italic letters indicate a statistically significant difference at a *P* < 0.05 as determined by ordinary 1-way ANOVA test. Scale bar = 1 cm. (c and d) Phenotypic analysis of root hair length in WT, RACK1A-GFP line, *rack1a-1*, *rack1a-5, fsd1-1* mutants, and *fsd1-1 rack1a-1* double mutant. (c) Representative pictures of 7-d-old seedlings mature root hairs measured at a distance of more than 5 mm from the root tip. Scale bar applies to all images and represents 500 μm. (d) Quantification of root hair length. Phenotypic analysis was performed in 3 repetitions (*N* = 1200). Violin plots represent the distribution of individual root hair length values. Lines represent the median value, and dashed lines represent quartile values. Different italic letters above columns indicate a statistically significant difference at a *P* < 0.05 as determined by Kruskal–Wallis 1-way analysis of variance on ranks.

Phenotypic analysis of *rack1a-1* lines complemented by RACK1A-GFP*, rack1a-1, rack1a-5, fsd1-1* single, and *fsd1-1 rack1a-1* double mutants showed genetic interaction between FSD1 and RACK1A affecting plant development (Fig. 4a–d).

The primary root length of 10-d-old WT, RACK1A-GFP and *fsd1-1* plants exhibited similar values. Conversely, *rack1a-1* and *rack1a-5* mutants were shorter in root length, and *fsd1-1 rack1a-1* double mutants exhibited the shortest primary roots (Fig. 4a and b). Therefore, *FSD1* and *RACK1A* show additive genetic influence in regulation of primary root length.

Both proteins exhibit accumulation in tips of growing root hairs, indicating possible genetic interaction in root hair growth regulation. To investigate this, we analyzed root hair length in all studied lines (Fig. 4c and d). In WT, the root hair length median value was 492 µm, with interquartile ranging from 376 µm to 591 µm. Comparably, the median values of root hair length in complemented line and *fsd1-1* mutant were 493 µm and 490 µm, respectively. Quartile values ranged from 398 µm to 580 µm in complemented line and from 397 µm to 586 µm in *fsd1-1* mutant, showing high similarity when compared to WT. On the contrary, root hair length in both *rack1a* mutants, as well as in *fsd1-1 rack1a-1* double mutant was significantly higher. While in *rack1a-1* mutant and *rack1a-5* mutant, the median values reached 582 µm (with quartiles ranging from 505 µm to 658 µm) and 570 µm (with quartiles ranging from 492 µm to 654 µm), respectively, in *fsd1-1 rack1a-1* double mutant, the median value was substantially lower with 519 µm. Additionally, the root hair length of *fsd1-1 rack1a-1* double mutant was significantly higher than the length measured in WT, complemented line and *fsd1-1* line, thus displaying an intermediate phenotype between *fsd1-1* and *rack1a-1* mutant (Fig. 4c and d). The results suggest a genetic interaction between FSD1 and RACK1A, involved in regulation of root hair growth and primary root development.

### RACK1A accumulation in SGs in response to salt stress

**Figure 5.**
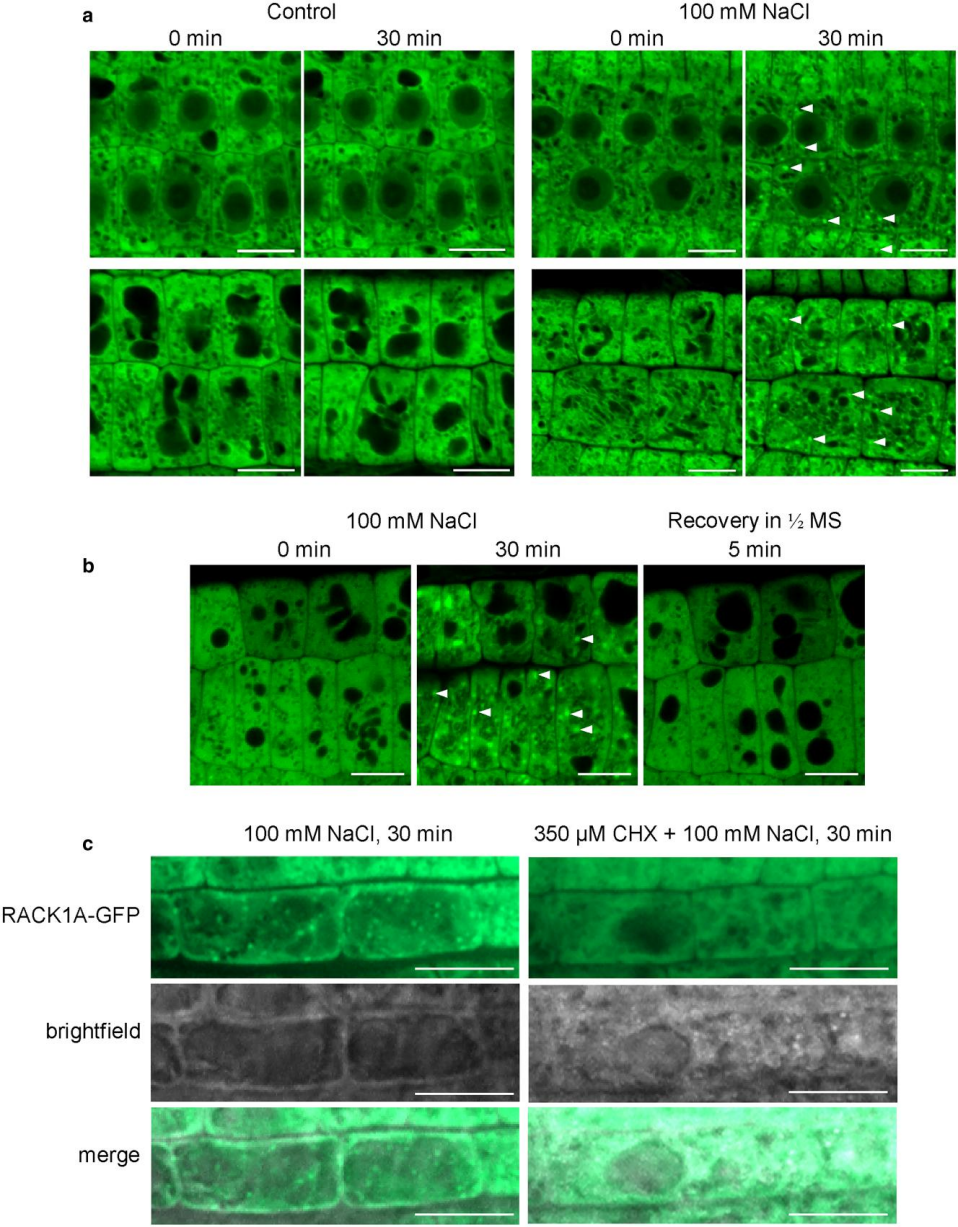
RACK1A-GFP localization in root meristematic zone cells upon 100 mM salt stress. (a) Root meristem cells before and 30 min after the treatment with ½ MS (Control) and 100 mM NaCl. Images were taken from middle focal plane containing nucleus and cytosol (upper part of the images) or upper focal plane containing cytosol near plasma membrane (bottom of the pictures). White arrowheads indicate a condensation of the fluorescence signal. Scale bars = 10 µm. (b) RACK1A-GFP localization in root meristematic zone cells after replacement of NaCl with liquid ½ MS medium. Images were taken from cortical focal plane. White arrowheads indicate a condensation of the fluorescence signal. Scale bars = 10 µm. (c) RACK1A-GFP localization in root epidermal cells after the treatment with 100 mM NaCl and 100 mM NaCl with 350 µM cycloheximide (CHX). Scale bars = 20 µm.

Although the roles of RACK1 in plant stress responses have been intensively studied, limited attention is devoted to the conditional localization of this protein. The above-mentioned interaction of RACK1A with SG marker proteins TSN1 and TSN2 suggests its relocation to these condensates. Therefore, we aimed to determine the changes in localization and intensity of the RACK1A-GFP signal in response to salt stress (Fig. 5). The meristematic cells of the root tip were imaged immediately after perfusion of the liquid media (*t* = 0 min) and again after 30 min. In control conditions, no change in the intensity or localization of the RACK1A-GFP signal was observed during the observation (Fig. 5a). Likewise, no changes were observed immediately after the addition of 100 mM NaCl (*t* = 0 min; Fig. 5a). On the other hand, incubation of seedlings with 100 mM NaCl for 30 min caused the formation of small, strongly fluorescent moving condensates, resembling SGs. These granular structures varied in size and were localized in the cytosol but not in the nucleus (Fig. 5a). Recovery from the salt stress by exchanging the NaCl-containing medium with control ½ MS medium resulted in the immediate disappearance of these SG-like structures (Fig. 5b). We also tested their formation under simultaneous effect of NaCl and SG inhibitor cycloheximide. Importantly, the presence of cycloheximide in the NaCl-containing medium prevented the formation of these structures, supporting the hypothesis about their identity (Fig. 5c). To further examine their identity, we transiently co-expressed the RACK1A-GFP construct with a SG marker *35S::RFP:RBP47* (Gutierrez-Beltran et al. 2021) in *N. benthamiana* leaf epidermal cells (Supplementary Fig. S10a). Salt stress triggered the accumulation of RACK1A-GFP in distinct cytoplasmic puncta, showing a high degree of colocalization with RFP-RBP47, as shown by Pearson’s and Manders’ colocalization coefficients (Supplementary Fig. S10b).

### Colocalization of RACK1A with FSD1

**Figure 6.**
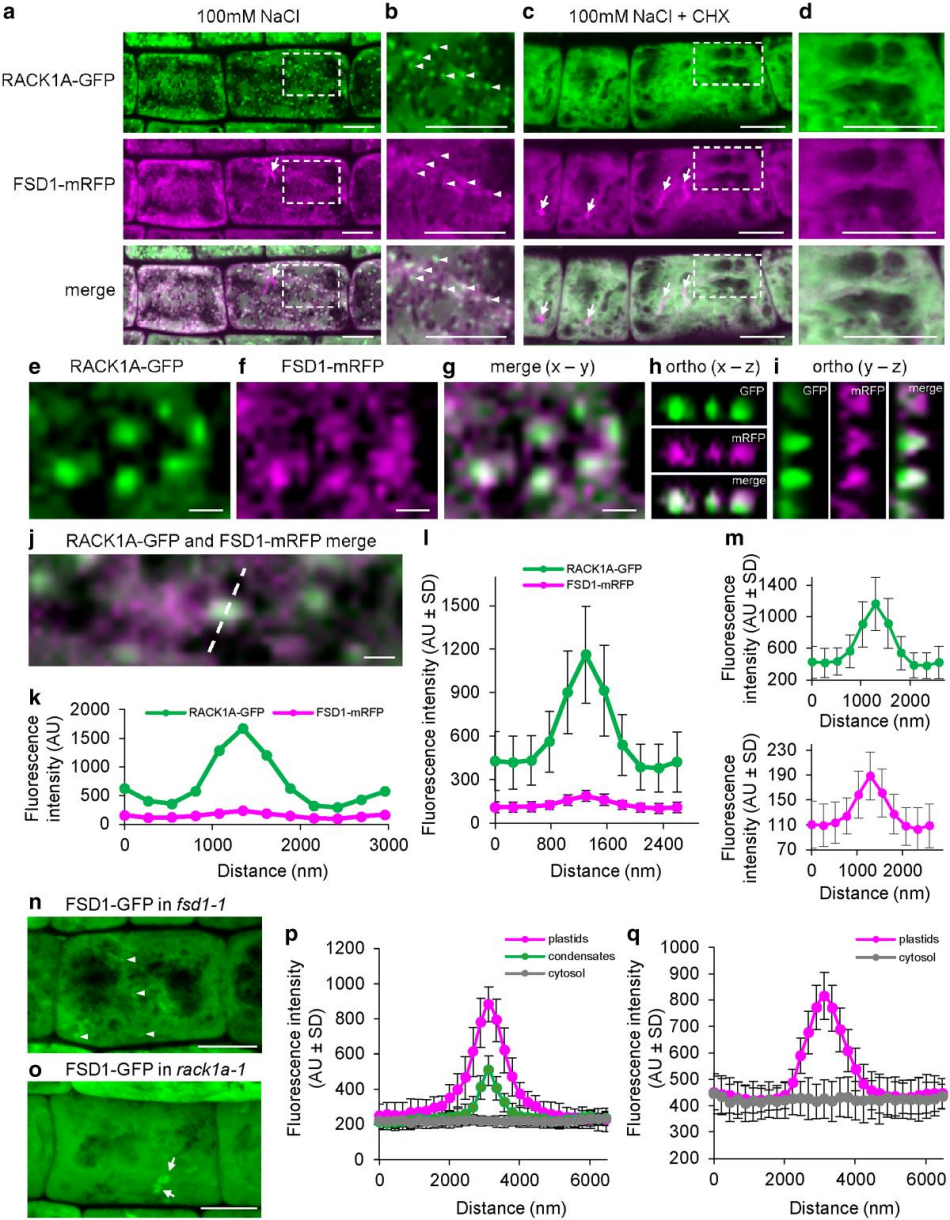
Subcellular localization analysis of RACK1A-GFP and FSD1-mRFP in root epidermal cells of the *rack1a-1* mutant after NaCl and cycloheximide (CHX) treatments. (a–d) Overview (a and c) and detailed view (b and d) of cortical cytoplasm in epidermal cells of the root transition zone of the genetically-rescued *rack1a-1* mutant showing distribution of RACK1A-GFP (green), FSD1-mRFP (magenta), and their overlay (merge) after treatment with ½ MS containing 100 mM NaCl (a and b) or 100 mM NaCl + 350 µM CHX (c and d) for 30 min. The detailed views (b and d) show close-ups marked by the dashed rectangle insets in (a and c). White arrowheads indicate structural condensates accumulating both GFP and mRFP fluorescence signals. (e–i) Qualitative colocalization analysis after treatment with ½ MS containing 100 mM NaCl for 30 min between RACK1A-GFP (e) and FSD1-mRFP (f) in an overlay image in a *x–y* view (g) and an orthogonal *x–z* (h) and *y–z* (i) spatial views in structural condensates from marked cell in (a). (j–m) Semi-quantitative colocalization analysis between RACK1A-GFP and FSD1-mRFP in a selected structural condensate (j) after treatment with ½ MS containing 100 mM NaCl for 30 min by analysis of fluorescence intensity (k) along the profile marked by a white line in (j). Averaged fluorescence intensity distribution from a profile measurement of structural condensates (*N* = 49) showed colocalization of GFP and mRFP fluorescence peaks (l), substantiated after their separate display with relevant range of fluorescence intensity distribution values (m). Arrows in (a and c) point FSD1-mRFP localized in plastids. (n–q) Semi-quantitative analysis of FSD1-GFP fluorescence signal distribution after 100 mM NaCl treatment (*N* = 20) in plastids, condensates, and cytosol of *fsd1* mutant (n and p) and in plastids and cytosol of *rack1-1* mutant (o and q). White arrowheads indicate structural condensates and arrows indicate plastids. Scale bars = 20 µm (a–d, n and o), 1 µm (e–g and j).

Next, we examined the colocalization of FSD1-mRFP and RACK1A-GFP fusion proteins in a rescued *rack1a* mutant line. In root epidermal cells, both proteins colocalized in cortical cytoplasm and, upon 30 min of salt treatment, showed colocalization in structural condensates resembling SGs, while both proteins still colocalized in the cytoplasm (Fig. 6a and b). Notably, cycloheximide treatment prevented this colocalization, as well as the formation of structural condensates (Fig. 6c and d). Colocalization of RACK1A-GFP with FSD1-mRFP in structural condensates was confirmed by qualitative (Fig. 6e–i) and semi-qualitative (Fig. 6j–m) colocalization analyses. Furthermore, FSD1-GFP colocalized with RFP-RBP47 in SGs after transient co-expression in salt-treated *N. benthamiana* leaves (Supplementary Fig. S11).

We performed a time-course analysis of RACK1A-GFP and FSD1-mRFP fluorescence in response to salt treatment, separately monitoring their localization in the SGs and the cytoplasm (Supplementary Fig. S12a and b). We have found that RACK1A-GFP fluorescence increased continuously over time in both SGs and the cytoplasm in response to NaCl, showing a more pronounced increase in SGs (Supplementary Fig. S12c). The FSD1-mRFP signal decreased within 10 min of NaCl treatment, followed by an increase in SGs, and colocalized with RACK1A-GFP. In contrast, the cytoplasmic FSD1-mRFP signal, which also colocalized with RACK1A-GFP, remained constant throughout this experiment (Supplementary Fig. S12d). Thus, these results indicate that both RACK1A-GFP and FSD1-mRFP signals increased in SGs, nevertheless, a considerable amount of both proteins remained in the cytosol. We also evaluated the number of SGs labeled by both RACK1A-GFP and FSD1-mRFP, showing that the FSD1-mRFP signal was present in almost 90% of RACK1A-GFP-positive SGs (Supplementary Fig. S12e).

### FSD1-GFP localization in *rack1a* mutant

To reveal putative RACK1A-mediated or RACK1A-controlled alterations in FSD1 subcellular localization after salt stress, we generated stably transformed *rack1a* lines expressing FSD1-GFP under its native promoter. The localization pattern of FSD1-GFP in *rack1a-1* mutant was compared with the complemented *fsd1-1* mutant (Dvořák et al. 2021). The observation of FSD1-GFP in *fsd1-1* and *rack1-1* mutants showed that while the signal of FSD1-GFP in *fsd1-1* mutant, in addition to its accumulation in plastids, was homogenously distributed in cortical cytoplasm in control conditions, FSD1-GFP accumulated in structural condensates upon 30 min salt treatment (Fig. 6n and p). Such accumulation of FSD1-GFP in structural condensates did not occur in *rack1a-1* mutant (Fig. 6o and q). Therefore, this genetic evidence suggests that RACK1A is indispensable for the condensate-specific FSD1 localization upon salt stress.

### Examination of FSD1 activity associated with SG-enriched fraction

**Figure 7.**
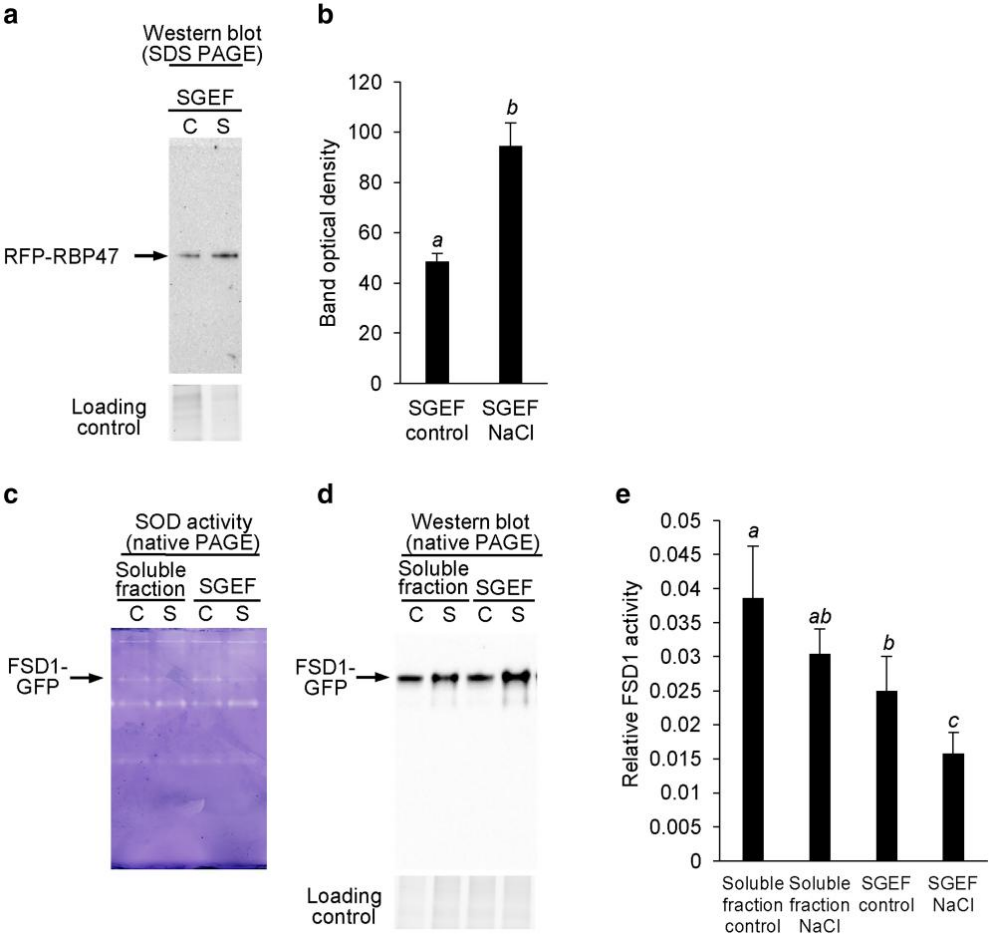
Examination of FSD1 activity in a stress granule-enriched fraction (SGEF) containing FSD1-GFP. *Nicotiana benthamiana* leaves were co-infiltrated with *Agrobacterium tumefaciens* harboring gene constructs for FSD1-GFP and RFP-RBP47 fusion proteins. After 2 d, excised leaves were treated with ½ MS with (salt, S) or without NaCl (control, C). SGEF was isolated and, together with the soluble extract subjected to denaturing (a) or native (c and d) polyacrylamide electrophoresis. The denaturing PAGE was used for immunoblot analysis using anti-mRFP antibody (a), and quantified in (b), while the native PAGE was used for in-gel SOD activity staining (c) and immunoblot analysis using anti-GFP antibody (d and e). The RFP-RBP47 abundance was expressed as quantification of the respective band optical density (OD). The FSD1 activity was expressed as a ratio of OD of the respective band (c) to the OD of a band corresponding to FSD1-GFP on the immunoblot (d). The experiment was repeated with 3 biological replicates. Different letters above the columns in (b) and (e) indicate a statistically significant difference as revealed by 1-way ANOVA with post-hoc Tukey HSD test (*P* < 0.05; mean ± SD).

We isolated an SGs-enriched fraction (SGEF) from *N. benthamiana* leaves transiently co-expressing FSD1-GFP and RFP-RBP47 following exposure to 200 mM NaCl. Immunoblot analysis of denatured protein extracts revealed an NaCl-induced increase in the abundance of the SG marker protein (Fig. 7a and b), consistent with enhanced SG formation under salt stress. Enzymatic assays showed a faint band corresponding to the activity of the FSD1-GFP fusion protein (Fig. 7c), which aligned with the chemiluminescent signal detected by immunoblotting with an anti-GFP antibody (Fig. 7d). When normalized to FSD1-GFP abundance, the activity of the FSD1-GFP fusion protein in the soluble extract did not change significantly upon salt treatment (Fig. 7e). In contrast, FSD1 activity in the SGEF fraction decreased under control conditions and was further reduced following salt treatment (Fig. 7e), suggesting that the FSD1 activity is lower in SGEF compared to the soluble extract.

### ROS levels are differently modulated in mutant and transgenic lines upon salt stress response

**Figure 8.**
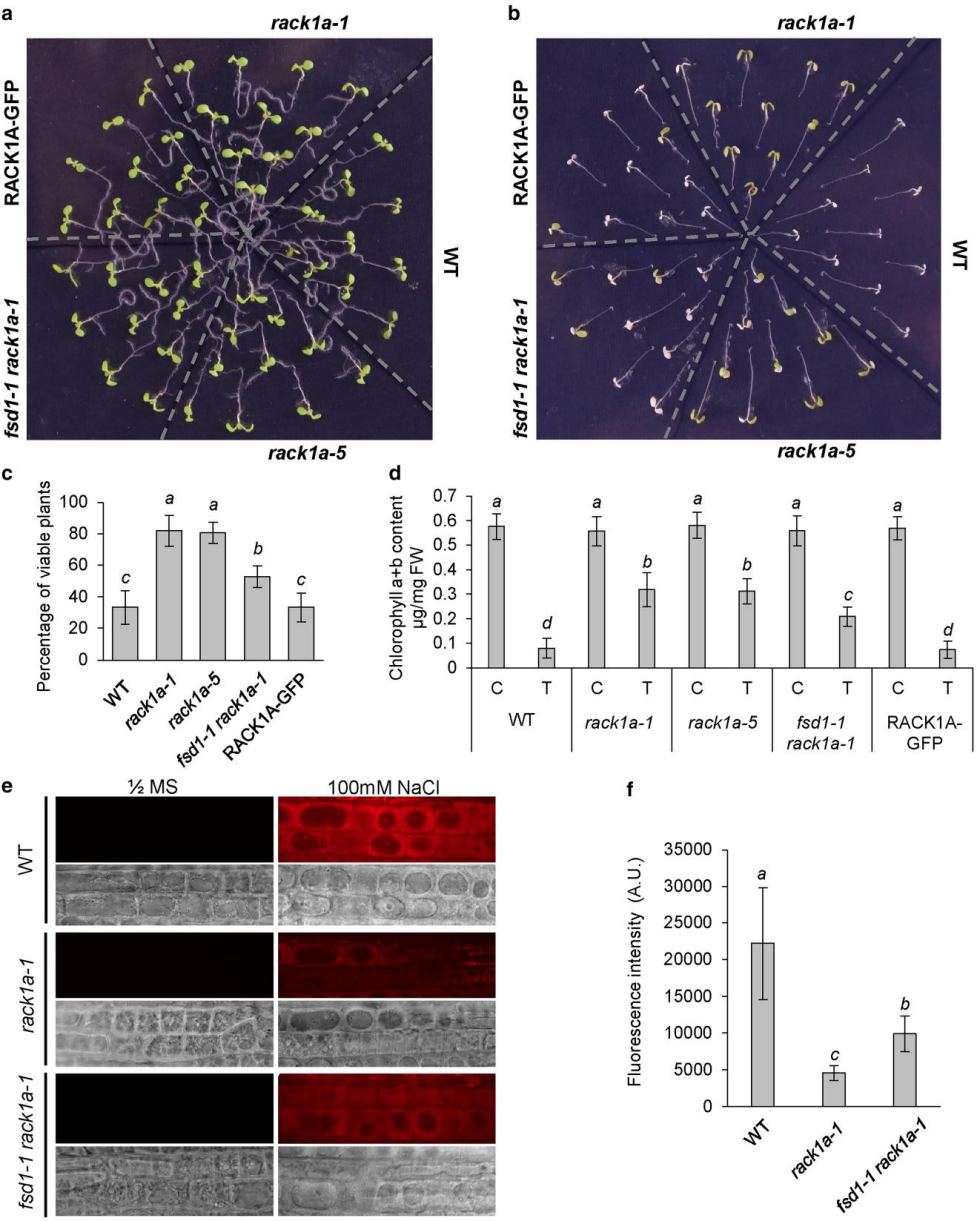
Analysis of NaCl-induced oxidative stress response in Col-0 (wild type; WT), *rack1a-1*, *rack1a-5* and *fsd1-1rack1a-1* mutants, and RACK1A-GFP line. Five-day-old seedlings were transferred to control and NaCl-containing medium. (a and b) Representative images of seedlings taken 5 d after the transfer to the control (a) and 150 mM NaCl-containing medium (b). (c) Quantification of seedlings with affected viability from (b). Plants with fully bleached cotyledons were considered unviable (mean ± SD; *N* = 90). (d) Quantification of chlorophylls a and b in control and salt-treated (150 mM NaCl) seedlings (mean ± SD; *N* = 40). (e) ROS distribution visualized by fluorescent tracker CellRox Deep Red reagent in plasmolyzed root epidermal cells of WT, *rack1a-1*, *fsd1-1 rack1a-1* lines. ROS accumulation in mock-treated (½ MS) and plasmolyzed root epidermal cells (½ MS with 100 mM NaCl) visualized by fluorescent tracker CellRox Deep Red reagent. The observed regions of root epidermal cells were visualized using transmitted light. Scale bars = 20 µm. (f) Quantification and statistical evaluation of CellRox Deep Red reagent fluorescence intensity (mean ± SD; *N* = 15). Different italic letters above the columns in (c, d), and (f) indicate a statistically significant difference between the respective lines and treatments at a *P* < 0.05 as determined by one-way ANOVA with post-hoc Tukey HSD test.

Previously, we determined the salt stress susceptibility of *fsd1* mutants, showing less viable plants after salt treatment compared to WT and both complemented FSD1-GFP and GFP-FSD1 lines (Dvořák et al. 2021). Here, results indicate an increased salt stress resistance of the single *rack1a-1* and *rack1a-5* mutants. The simultaneous absence of FSD1 and RACK1A leads to a salt stress response intermediate between WT and the single mutants (Fig. 8a–c). These results were also corroborated by chlorophyll content measurement (Fig. 8d). Our data indicate that *FSD1* is epistatic to *RACK1A* in salt stress response, and both genes jointly modulate the salt stress responses in Arabidopsis.

Finally, we also examined the intracellular ROS levels in root cells with the vital fluorescent dye CellRox Deep Red, which labels superoxide and hydroxyl radicals. As shown previously, the hypersensitive *fsd1* single mutants showed higher fluorescence compared to WT in response to salt stress, indicating more intensive ROS accumulation in root cells (Dvořák et al. 2021). This vital histochemical staining showed significantly weaker fluorescence in roots of *rack1a* mutants as compared to WT (Fig. 8e and f). The *fsd1-1 rack1a-1* double mutant showed intermediate CellRox Deep Red fluorescence in response to salt (Fig. 8e and f), while a statistically significant difference was observed between the single and double mutants. Collectively, the presented data support the regulatory role of the RACK1A-FSD1 module in Arabidopsis salt stress responses, likely via ROS levels regulation.

## Discussion

Previous studies suggested the involvement of RACK1 in salt stress responses in different plant species (Guo et al. 2009; Li et al. 2017 ; Zhang et al. 2018; Denver and Ullah 2019). However, the subcellular localization of RACK1A upon salt stress has not been reported so far. In this study, we observed that RACK1A-GFP accumulates in SGs upon salt stress. As shown previously, SG marker protein TSN1 localizes to SGs upon salt stress, and *TSN1* RNAi lines exhibit higher salt stress sensitivity (Yan et al. 2014). Both TSN1 and TSN2 were found in the RACK1A interactome in this and another very recent study (Zheng et al. 2025), while we also proved the interaction between RACK1A and TSN1 by rBiFC assay. The comparison of interactomes of RACK1A (this study) and TSN1/2 (Gutierrez-Beltran et al. 2021) shows numerous common proteins, involved in mRNA stability, ABA signaling, cell wall regulation, gene expression and membrane trafficking (Supplementary Dataset S5). Thus, RACK1A and TSN1/2 likely cooperate in the regulation of plant responses to salt stress. Of note, the participation of RACK1A in salt stress response cannot be assigned solely to its accumulation in SGs, because a considerable pool of RACK1A remains in the cytosol. Mammalian RACK1 was identified as an SG component, and its sequestration to these condensates determines the cell fate upon diverse stress stimuli (Park et al. 2020; Masi et al. 2022). Nevertheless, the accumulation of plant RACK1A in SGs has not been reported so far.

Our study also uncovered a previously unknown, SG-mediated mechanism of antioxidant enzyme regulation, demonstrating that FSD1, an important superoxide decomposing enzyme, accumulates in SGs during salt stress. Notably, previous proteomic studies proposed the SG accumulation of FSD1 (Kosmacz et al. 2019). Our findings demonstrate that RACK1A likely mediates the recruitment of a distinct pool of FSD1 to SGs, while the FSD1 activity remains lower compared to the soluble fraction. This is in line with the expected increase in ROS levels during salt stress response. Thus, RACK1A likely utilizes FSD1 to amend the superoxide/H_2_O_2_ ratio in response to salt stress, perhaps by modulating salt stress signaling. The precise role of the RACK1A-FSD1 module in stress granules remains unknown. However, electrochemical measurements provided evidence about ROS presence in these condensates (Hu et al. 2021).

Our interactome, together with previously published studies (Guo et al. 2019), suggests SOD isoforms CSD1, CSD2, MSD1, FSD2, and FSD3 as potential interaction partners of RACK1A. The crosstalk between CSDs and RACK1A appears more complex, largely because RACK1A promotes the biogenesis of miRNA398, which directly downregulates both *CSD1* and *CSD2* mRNAs (Speth et al. 2013). In *rack1a* mutants, this regulatory mechanism is likely disrupted, leading to increased abundance and activity of CSD isoenzymes. Therefore, the functional significance of the RACK1A–CSD interaction may differ from that of other isoforms. Additionally, interaction with the chloroplastic (CSD2, FSD2, FSD3) and mitochondrial (MSD1) isoforms would only occur if they were mislocalized to the cytoplasm.

Further investigations will determine whether RACK1A may participate in the coordination of superoxide and H_2_O_2_ scavenging machinery in plants. Identification of H_2_O_2_-decomposing enzyme ASCORBATE PEROXIDASE 1 (APX1), which is a potential RACK1A interactor (Guo et al. 2019), in SGs (Kosmacz et al. 2019) supports this assumption. Taken together, apart from CPN20 (Kuo et al. 2013) and WRKY53 (Andrade Galan et al. 2024), RACK1A may serve as a negative regulator of FSD1 activity.

We have also found that RACK1A functions jointly with FSD1 to regulate root hair tip growth. Root hair development consists of a bulge formation and subsequent polarization of the cytoskeleton, membrane trafficking, and cell wall deposition at the tip to ensure polar expansion and root hair growth (Šamaj et al. 2006; Kuběnová et al. 2022). Apoplastic and apical cytoplasmic ROS are essential factors participating in root hair elongation (Schoenaers et al. 2017; Kuběnová et al. 2023). Peroxidases and *RESPIRATORY BURST OXIDASE HOMOLOG* (*RBOH*) family of enzymes (Torres et al. 2002; Sagi 2006; Kuběnová et al. 2022) participate in apoplastic ROS formation. RBOHs at the plasma membrane release superoxide to the apoplast, which is decomposed to H_2_O_2_, entering the cytoplasm through aquaporins (Gerber et al. 2025). During root hair elongation, H_2_O_2_ accumulates in the apical region of the root hair. Root hairs, however, also show prominent mitochondrial accumulation of ROS (Kuběnová et al. 2023). RACK1A-GFP accumulated in the tip of growing root hairs, similar to FSD1-GFP, as reported previously (Dvořák et al. 2021). Notably, knock-out double mutant in *RACK1A* and *FSD1* showed partial rescue of longer root hair phenotype typical for *rack1a* single mutants. Therefore, FSD1 might be an important factor modulating root hair elongation by positively affecting polar distribution of cytoplasmic ROS in the *rack1a* mutant, supporting the H_2_O_2_ production in the apical region. Thus, RACK1A likely conditionally alters the FSD1 activity to modify cytosolic ROS distribution during root hair elongation. The enhanced root hair elongation may, together with elevated FSD1 activity, facilitate the salt stress tolerance of the *rack1a* mutants by improving water uptake. In agreement, *OsRACK1A*-suppressed transgenic rice maintains low Na^+^/K^+^ concentrations during salt stress (Zhang et al. 2018) and exhibits elevated drought stress tolerance (Li et al. 2009).

In summary, we identified RACK1A as a protein partially accumulating in SGs upon salt treatment. It facilitates the recruitment of a distinct pool of FSD1 to SGs during salt stress and reduces its activity. The absence of RACK1A leads to FSD1 activation, preventing its SG-specific accumulation and higher salt stress resistance of seedlings. The RACK1A-FSD1 module is also involved in developmental programs such as root hair elongation. These results reveal a mechanism of salt stress response in Arabidopsis.

## Materials and methods

### Plant material and growth conditions

*Arabidopsis thaliana* (L.) Heynh ecotype Columbia (Col-0), referred to as wild-type (WT), *fsd1-1* and FSD1-GFP line (Dvořák et al. 2021), *rack1a-1* (Chen et al. 2006), *rack1a-5* (CRISPR/Cas9-mediated insertion mutant line prepared in this study), and *fsd1-1 rack1a-1* (prepared by conventional crossing in this study) mutant lines (all in the Col-0 background) have been used in experiments. We also generated transgenic *rack1a-1* bearing *5′UTR-pRACK1A::gRACK1A:GFP:3′UTR* (hereafter referred to as RACK1A-GFP line), which was afterwards transformed by the floral dip method with *5′UTR-pFSD1::gFSD1:mRFP:3′UTR* construct. Additionally, the *5′UTR-pFSD1::gFSD1:GFP:3′UTR* (referred to as FSD1-GFP) construct from Dvořák et al. (2021) was transformed into the *rack1a-1* mutant. Selected lines with one insertion were propagated into the T3 homozygous generation.

Seeds were surface-sterilized by ethanol, dried, and placed on a half-strength Murashige and Skoog (½ MS) medium and grown at 21 °C and 70% humidity under a 16 h light/8 h darkness photoperiod with a photosynthetic photon flux of 120 μmol·m^2^·s^−1^ in an environmental chamber (Weiss Technik, Grand Rapids, MI, United States) provided by cool white fluorescent linear tube light sources (Philips Master TL-D Reflex 36 W, light flow 3350 lm, light efficiency 93 lm·W^–1^) for a maximum of 14 d. Additionally, wild-type *Nicotiana benthamiana* plants were grown from seeds in pots with soil for 5 weeks under the same conditions as described above.

### CRISPR/Cas9-mediated *RACK1A* mutagenesis

We employed CRISPR/Cas9-mediated mutagenesis (Decaestecker et al. 2019) to prepare a knock-out *rack1a* mutant. Oligomers encoding *RACK1A*-specific guide-RNAs (gRNAs) were designed using CRISPR-P v2.0 (Liu et al. 2017) (Supplementary Table S1), synthesized and annealed. Entry (containing gRNA scaffold sequence, *AtU6* promoter sequence, and one of 4 annealed gRNAs) and destination vectors (containing *Ubi10::Cas9:mCherry*, *OLE1:mRuby* and all 4 *RACK1A*-specific *gRNAs*) were prepared by GoldenGate cloning (Engler et al. 2008). Expression vectors were introduced into *Agrobacterium tumefaciens* GV3101 and used for floral dip of WT plants. T1 generation seeds were selected by red fluorescence for the presence of *OLE1:mRuby* using a stereomicroscope (Axio Zoom.V16; Carl Zeiss, Germany). Random T2 generation plants expressing *Cas9:mCherry* were selected by stereomicroscope and gRNA1- or gRNA2-mediated mutations were detected by restriction fragment length polymorphism (RFLP) analysis in a 1793 bp sequence (containing all 4 gRNA complementary sequences) that was amplified using specific primers (Supplementary Table S2). Next, T3 generation plants not expressing *Cas9:mCherry* were selected by fluorescence microscopy and subjected to RFLP. The PCR products of selected plants were sequenced by Sanger sequencing using specific primers (Supplementary Table S2) and analyzed by DECODR (Bloh et al. 2021) and ICE CRISPR Analysis Tool (Conant et al. 2022) software. Finally, homozygous insertion mutant plants were selected in the T4 generation by sequencing.

### Preparation of transgenic *rack1a-1* line complemented with GFP-tagged RACK1A under control of native promoter

Genomic DNA of *RACK1A* from WT, including native promoter sequence, *5′UTR* and *3′UTR* sequences, were used to prepare the fusion construct with a gene encoding *5′UTR-pRACK1A::gRACK1A:GFP:3′UTR* (referred to as RACK1A-GFP). Specific primers (Supplementary Table S2) were used to cover the sequence 1,483 bp upstream of the start codon and 498 bp downstream of the stop codon. MultiSite Gateway Three-Fragment Vector Construction (Thermo Fisher Scientific) kit was used to prepare the constructs. Briefly, PCR-amplified DNA of *5′UTR*, *RACK1A* gDNA and *3′UTR* were introduced into pDONRP4-P1R (A fragment) or pDONRP2R-P3 (C fragment) donor vectors by BP reaction to produce entry vectors. Constructs were introduced into *Escherichia coli* TOP10 strain and positive clones were selected. Three-fragment LR recombination reaction was used to clone the fragments into the destination vector pB7m34GW. Positive clones were selected and confirmed by sequencing. T1 generation seeds were grown on a plant selection medium containing 10 μg·mL^−1^ phosphinothricin. Transgenic *rack1a-1* lines carrying RACK1A-GFP were propagated into T3 generation to ensure the presence of the transgene in both alleles.

### Phenotypic analyses of the mutant lines

Ten-day-old seedlings were documented for primary root length measurement using a flatbed scanner (ImageScanner III). The length of primary roots was measured by ImageJ (Schneider et al. 2012). Axio Zoom.V16 microscope was used to image root hairs and Zen Blue 2012 software (Carl Zeiss, Jena, Germany) was used to measure root hair length. The root phenotypic analysis was performed in 3 biological replicates. Fifteen plants per replicate were used for primary root length measurements. At least 300 root hairs from 20 plants were measured per replicate for root hair analysis. Mature root hair phenotypes were assessed in 7-d-old seedlings at a specifically designated distance from the primary root tip, ensuring measurements were taken more than 5 mm away. Statistical significance was evaluated using a 1-way ANOVA test in GraphPad Prism 8.3.0 (GraphPad Software, San Diego, CA, USA).

### Preparation of recombinant RACK1A

A restriction-based cloning was employed to generate gene constructs encoding recombinant RACK1A fused to glutathione S-transferase (GST). The cDNA of *RACK1A* was amplified by PCR using primers (Supplementary Table S2) containing the appropriate restriction sites (BamHI, EcoRI). The PCR product and the *pGEX-6P-1* vector (Merck, GE28-9546-48) were digested with the corresponding restriction enzymes and ligated. Positive clones were verified by restriction digestion and confirmed by sequencing. The final expression plasmid was transformed into *E. coli* BL21 Star (DE3) competent cells. Expression and purification of recombinant protein was performed according to the protocol specified in the instruction manual of the pGEX vector, and the quality of purification was examined by SDS-PAGE with Coomassie blue staining.

### Immunoblotting and in-gel SOD activity analysis

To prepare protein extract, root homogenate (100 mg) was resuspended in 200 μL of extraction buffer (50 mM sodium phosphate (pH 7.8), 10% (v/v) glycerol, 2 mM ascorbate), placed on ice for 30 min, and occasionally vortexed. The extract was centrifuged at 13,000 × g for 20 min at 4 °C, and the protein concentration of the supernatant was measured according to the Bradford method (Bradford 1976). The protein extract was used for the in-gel SOD activity assay and total SOD activity measurement in the presence of recombinant GST-RACK1A. For immunoblot analysis, native protein extracts were supplemented with 4 times concentrated Laemmli SDS buffer at a 3:1 ratio and 5% (v/v) β-mercaptoethanol. The samples were boiled for 5 min at 95 °C before SDS-PAGE electrophoresis.

The immunoblotting and in-gel activity analyses were performed according to Melicher et al. (2022). Anti-FeSOD (AS06125, Agrisera, Sweden), anti-CSD2 (AS06170, Agrisera, Sweden) and anti-RACK1A (AS111810, Agrisera, Sweden) primary antibodies were used for immunoblotting in 1:1,000, 1:2,000 and 1:3,000 dilutions, respectively. As stated in the datasheet, the anti-CSD2 antibody recognizes CSD1 and CSD2 isozymes.

### SOD activity measurement in the presence of GST-RACK1A

The total SOD activity was measured using a colorimetric method with the SOD activity assay kit (Merck, CS0009) according to the manufacturer's protocol with slight modification. In brief, 20 μL of root protein extract, prepared as described above, containing 1.5 μg of proteins, was incubated with or without recombinant GST-RACK1A at ratios of 1:1 and 1:10, in terms of protein content, at 25 °C for 1 h. The solution was added to the final reaction mixture (total volume 200 μL) to initiate the reaction. The measurements were performed in 3 biological replicates.

### Evaluation of salt stress responses

To determine the sensitivity of plants to salt stress, 4-d-old seedlings of WT, *fsd1-1*, *rack1a-1*, *rack1a-5*, *fsd1-1 rack1a-1* mutants and RACK1A-GFP-complemented *rack1a-1* mutant growing on ½ MS medium were transferred to the same medium containing 200 mM NaCl. The number of viable and fully bleached seedlings was counted on the fourth day after transfer to NaCl-containing media and documented using a digital camera (NIKON Inc., Tokyo, Japan). Measurements were performed in 3 biological replicates with 30 plants per replicate.

The relative amounts of chlorophyll a and b were measured according to Melicher et al. (2024). Chlorophyll concentrations were determined from 10 seedlings per line (total of 40 seedlings analyzed). The contents of chlorophyll a and b across all replicates were normalized to fresh weight, plotted, and statistically evaluated using 1-way ANOVA followed by Tukey's HSD *post hoc* test.

### LSFM

The plant material (RACK1A-GFP) preparation, mounting the seedling to fluorinated ethylene propylene tube (Wolf-Technik, Germany) and its insertion into the observation chamber of the light-sheet microscope were performed as described previously by Ovečka et al. (2015). After 30 min of sample stabilization, developmental live cell imaging of the primary root was done by Zeiss Light-sheet 7 (Carl Zeiss, Germany) with dual-side illumination using two 10×/0.2 NA illumination objectives (Carl Zeiss, Germany) and pivot scan mode, excitation laser line 488 nm, beam splitter LP560, and emission filter BP505-545, detected with Plant-Apochromat 10×/0.5 numerical aperture (NA) water immersion objective (Carl Zeiss, Germany) and recorded by the PCO.Edge 4.2 camera (exposure time 20 ms). Time-course imaging was carried out in 5 consequential frames (Tiles) with an imaging frequency of every 1 min in Z-stack mode for 2 to 10 h. Developmental live cell imaging of lateral root formation was performed similarly by Zeiss Light-sheet Z.1 (Carl Zeiss, Germany), with single-side illumination using 10×/0.2 NA illumination objective and Plan-Apochromat 20×/1.0 NA water immersion detection objective (Carl Zeiss, Germany) with an exposure time of 100 ms and imaging frequency of every 10 min in the Z-stack mode for 12 to 17 h. Stitching of tiles was done in Zeiss ZEN 3.4 (Blue version) software and data post-processing in Zeiss ZEN 3.4 (Blue version) or ArivisVision4D 3.0.1 software (Arivis AG, Rostock, Germany). Exported images were assembled into final figure plates in Microsoft PowerPoint.

### CLSM and image analysis

Live-cell imaging was performed using a CLSM on Zeiss LSM710 (Carl Zeiss, Germany), Zeiss LSM880 equipped with an Airyscan detector (ACLSM, Carl Zeiss, Germany) or Cell Observer SD Axio Observer Z1 spinning disk microscope (Carl Zeiss, Germany). Image acquisition was performed with Plan-Apochromat 20×/0.8 NA M27 (Carl Zeiss, Germany) and Plan-Apochromat 40×/1.4 NA Oil DIC (Carl Zeiss, Germany). A 488 nm excitation laser, set at 4% of the available intensity, was used for EGFP detection. Using CLSM, the emission of EGFP was detected in the 490 to 560 nm range. Emission ranging from 495 to 550 nm was used for ACLSM imaging of EGFP. For mRFP detection, a 561 nm excitation laser line was used, with emission detected between 590 and 650 nm.

To analyze localization changes in response to salt stress, seedlings of 4-d-old RACK1A-GFP line were placed in liquid ½ MS media between a glass slide and a cover slip. First, cells from the root differentiation and elongation zones were imaged to examine the RACK1A-GFP localization under normal conditions. To induce salt stress, 100 mM NaCl in ½ MS was added to the seedlings in slides by perfusion. The RACK1A-GFP fluorescence was imaged for different time periods (30 min or 45 min). Recovery from salt stress was facilitated by replacing the solution with ½ MS medium without salt through perfusion. Cycloheximide (Sigma-Aldrich, C7698) treatment was performed using a 350 µM concentration (dissolved in distilled water) in liquid ½ MS medium with or without 100 mM NaCl.

ROS were visualized in root cells by incubating the samples in 30 μM CellROX Deep Red Reagent (Thermo Fisher Scientific), as described previously (Dvořák et al. 2021). The emitted fluorescence signal, excited at 639 nm, was captured within the 652 to 713 nm range using an Axio Observer.Z1 Spinning disk fluorescence microscope (Carl Zeiss, Germany).

Images with CellROX Deep Red Reagent signal were processed as single planes using Zen Blue 2012 software (Carl Zeiss, Jena, Germany), exported and adjusted in Microsoft PowerPoint to final figures. The signal was analyzed using ImageJ software. Images were converted to an 8-bit grayscale format, and the mean signal density was measured.

Kymographs of growing root hairs were generated from time-lapse images acquired by LSFM along the line drawn longitudinally in the central part of growing root hairs from maximum intensity projections using the Zen software (Blue version). Semi-quantitative fluorescence signal intensity analysis was performed by a profile function in one central Z-stack of the selected compartments. Profiles for signal intensity distribution and quantitative evaluation were performed in Zen Blue 2014 software.

### rBiFC assay-cloning and analysis

Arabidopsis total cDNA pool was used to amplify full-length cDNAs encoding *RACK1A, FSD1, TSN1, NUDIX HYDROLASE 7* (*NUDT7*), and *GUANINE NUCLEOTIDE-BINDING PROTEIN ALPHA-1 SUBUNIT* (*GPA1*) using specific primers (Supplementary Table S2). Preparation of expression vectors was performed using “2in1” cloning system as described in Grefen and Blatt (2012). MultiSite Gateway Three-Fragment Vector Construction (Thermo Fisher Scientific) kit was used to prepare the constructs. Expression vectors, carrying *RACK1A* fused to the N-terminal part of *YFP* (*YFPn*) as well as *FSD1*, *TSN1*, *NUDT7*, or *GPA1* fused to the C-terminal part of *YFP* (*YFPc*), were prepared.

*N. benthamiana* leaf epidermal cells transiently transformed with *A. tumefaciens*, carrying the above-mentioned vectors were used for semiquantitative rBiFC. Confocal imaging was performed on Zeiss LSM710 (Carl Zeiss, Germany) with Plan-Apochromat 20×/0.8NA (Carl Zeiss, Germany) objective. A 514 nm excitation laser was used for YFP imaging, with emission detected in the 520 to 580 nm range. For mRFP detection, a 561 nm excitation laser was used, with emission detected between 600 and 680 nm.

Images were processed as maximum intensity projections of acquired Z-stacks using Zen Blue 2012 software. RACK1A-NUDT7 (Olejnik et al. 2011) and RACK1A-GPA1 (Cheng et al. 2015) pairs were used as positive and negative controls, respectively. The relative abundance of interaction was calculated as a ratio of the mean fluorescence intensity of YFP relative to mRFP. The experiment was performed twice, and at least one region of interest (ROI) from 30 imaged cells per replicate was considered for calculations. The threshold ratio, accounting for background YFP fluorescence, was calculated from the analysis of negative control samples. Positive control was used to measure the ratio of known interactions, and the calculated average value was set to 1 for normalization. Statistical significance between studied interactions and negative control was evaluated using a 1-way ANOVA test in GraphPad Prism 8.3.0.

### Co-immunoprecipitation of RACK1A-GFP interacting proteins

The 14-d-old seedlings (2 g in fresh weight) of RACK1A-GFP line were treated with liquid ½ MS media with or without 150 mM NaCl for 1 h and ground in liquid nitrogen. The protein extraction, co-immunoprecipitation, and “on beads” trypsin digestion were performed as described in Hunter et al. (2019) and the peptides were cleaned on C18 cartridges (Bond Elut C18, 100 mg, 1 mL, Agilent Technologies). The LC-ESI-MS/MS analyses were performed on a nanoflow HPLC system (Easy-nLC1200, Thermo Scientific) coupled to the Q Exactive HF mass spectrometer (Thermo Scientific, Bremen, Germany). The peptide separation and data acquisition were performed as described in Hunter et al. (2019), while using Thermo Xcalibur 4.1 software (Thermo Fisher Scientific) for the data acquisition.

Raw data files were searched for protein identification using Proteome Discoverer 3.2 software (Thermo Scientific) connected to an in-house server running the Mascot 3.0.0 software (Matrix Science). Database search was performed against SwissProt database (version 2024_03) with taxonomy filter for *Arabidopsis thaliana*. The experiment was performed in 4 biological replicates. Proteins identified by one peptide and proteins detected only in one biological replicate were excluded. Proteins interacting with YFP (Hunter et al. 2019) were considered unspecific and were removed.

### Salt stress-induced colocalization of RACK-GFP and FSD1-GFP with the SG marker RFP-RBP47 in *N. benthamiana*

Five-week-old *N. benthamiana* leaves were infiltrated with *A. tumefaciens* strain GV3101 carrying P19, RACK1A-GFP and *35S::RFP:RBP47* (RNA-BINDING PROTEIN 47; Gutierrez-Beltran et al. 2021). Alternatively, FSD1-GFP was co-infiltrated with *35S::RFP:RBP47*. After 2 d of incubation, the transiently transformed leaves were used for microscopic analysis. Prior to microscopy, the excised infiltrated leaves were incubated in liquid ½ MS medium either with or without 200 mM NaCl for 1 h. Quantitative co-localization analysis between RACK1A-GFP or FSD1-GFP and RFP-RBP47 was conducted in particular ROIs corresponding to SGs structures. The measured area of SGs was selected manually with the drawing tool of the ZEN software. The colocalization range was measured from single plane confocal sections. In total, 70 independent SG structures were analyzed using the colocalization tool of Zeiss ZEN 2014 software (Blue version). Background thresholds were automatically implemented by the iterative Costes approach (Costes et al. 2004), and colocalization data were calculated from manually selected ROIs. Data were displayed in intensity-corrected scatterplot diagrams, the intensity correlation of colocalizing pixels was expressed as the average of Pearson's and Manders’ correlation coefficient using Microsoft Excel.

### Preparation of SG-enriched fraction

Leaves of *N. benthamiana* were co-infiltrated with *A. tumefaciens* harboring gene constructs encoding FSD1-GFP and RFP-RBP47 fusion proteins. After 48 h, the infiltrated leaves were incubated in liquid ½ MS medium, either with or without 200 mM NaCl for 1 h. The leaves were then homogenized in liquid nitrogen, and the homogenates were incubated with lysis buffer (50 mM Tris-HCl, pH 7.4). After centrifugation, an aliquot of the supernatant was retained for biochemical analyses (soluble extract), while the remaining fraction was used for the isolation of SG-enriched fractions according to Kosmacz and Skirycz (2020). Prior to biochemical analyses, the lysis buffer was substituted with 50 mM Na-phosphate buffer (pH 7.8) using Amicon ULTRA centrifugal filters (Merck) according to the manufacturer's instructions, and the protein concentrations were measured in the soluble extracts and SG-enriched fractions. Subsequently, SOD activity was visualized on native acrylamide gels as described above. A parallel native gel was subjected to immunoblot analysis and the PVDF membrane was probed with anti-GFP antibody (1:2,000; G1546; Sigma-Aldrich). Na-phosphate buffered protein extracts were denatured by the addition of 4 × Laemmli buffer and boiled for 5 min. Finally, the extracts were separated on denaturing PAGE and the proteins were transferred to PVDF membrane, which was probed with anti-mRFP antibody (1:2,000; R10367; Invitrogen). The activity of FSD1 was expressed as an optical density of the band corresponding to FSD1 activity relative to the optical density of the band corresponding to FSD1-GFP abundance. The experiment was carried out in 3 biological replicates.

### Accession numbers

Sequence data from this article can be found in the GenBank/EMBL data libraries under accession numbers: FSD1 (AT4G25100; NP_849440.1), CSD1 (AT1G08830; NP_172360.1), CSD2 (AT2G28190; NP_565666.1), RACK1A (AT1G18080; NP_173248.1), TSN1 (AT5G07350; NP_196352.2), and RBP47 (AT3G19130; NP_188544.1).

## Supplementary Material

kiaf659_Supplementary_Data

## Data Availability

The data underlying this article are available in the article and in its online supplementary material. Source files are available from the corresponding authors upon request.

## Supplementary material

The following materials are available in the online version of this article.

**Supplementary Figure S1.** Localization of RACK1A-GFP in 5-day-old seedling as observed using fluorescence ZOOM stereomicroscope.

**Supplementary Figure S2.** Microscopic observation of RACK1A-GFP subcellular localization during root hair development and tip growth.

**Supplementary Figure S3.** Time-course imaging of RACK1A-GFP localization and accumulation during root hair formation.

**Supplementary Figure S4.** Protein interaction networks assigned to the interactome of RACK1-GFP after mock treatment by STRING analysis.

**Supplementary Figure S5.** Protein interaction networks assigned to the interactome of RACK1-GFP after salt treatment by STRING analysis.

**Supplementary Figure S6.** Gene ontology annotation enrichment analysis of proteins identified by co-immunoprecipitation analysis of RACK1A-GFP

**Supplementary Figure S7.** Functional validation of RACK1A – TSN1 interaction.

**Supplementary Figure S8.** Validation of *rack1a-5* and *fsd1-1 rack1a-1* mutants by immunoblotting.

**Supplementary Figure S9.** Production, purification, and detection of recombinant GST-RACK1A.

**Supplementary Figure S10.** Salt stress induced colocalization of RACK1A-GFP and stress granule marker RFP-RBP47 in transiently transformed *N. benthamiana* epidermal leaf cells.

**Supplementary Figure S11.** Salt stress induced colocalization of FSD1-GFP and stress granule marker RFP-RBP47 in transiently transformed *N. benthamiana* epidermal leaf cells.

**Supplementary Figure S12.** Time course monitoring of NaCl-dependent changes in RACK1A-GFP and FSD1-mRFP fluorescence in *rack1a* mutant expressing *proRACK1A::RACK1A:GFP* and *proFSD1::FSD1:mRFP* constructs.

**Supplementary Table S1.** Oligomers encoding RACK1A-specific gRNAs used in CRISPR/Cas9-mediated RACK1A mutagenesis.

**Supplementary Table S2.** List of used primers.

**Supplementary Dataset S1.** List of proteins identified by co-immunoprecipitation analysis of RACK1A-GFP after control treatment.

**Supplementary Dataset S2.** List of proteins identified by co-immunoprecipitation analysis of RACK1A-GFP after salt treatment.

**Supplementary Dataset S3.** Gene ontology analysis of proteins identified by co-immunoprecipitation analysis of RACK1A-GFP after control treatment.

**Supplementary Dataset S4.** Gene ontology analysis of proteins identified by co-immunoprecipitation analysis of RACK1A-GFP after salt treatment.

**Supplementary Dataset S5.** List of proteins found commonly in interactomes of RACK1A (this study) and TSN1/2 (Gutierrez-Beltran et al., 2021).

**Supplementary Video S1.** Time-lapse imaging of primary root growth of RACK1A-GFP lines using LSFM.

**Supplementary Video S2.** Time-lapse imaging of developing root hairs in the root of RACK1A-GFP line using LSFM.

**Supplementary Video S3.** Time-lapse imaging of primary root growth of RACK1A-GFP lines using LSFM in a pseudo-color-coded semi-quantitative fluorescence intensity distribution.

**Supplementary Video S4.** Time-lapse imaging of developing root hairs in the root of RACK1A-GFP line using LSFM in a pseudo-color-coded semi-quantitative fluorescence intensity distribution.

**Supplementary Video S5.** Time-lapse imaging of developing lateral root primordia in the root of RACK1A-GFP line using LSFM.

**Supplementary Video S6.** Time-lapse imaging of developing lateral root primordia in the root of RACK1A-GFP line using LSFM in a pseudo-color-coded semi-quantitative fluorescence intensity distribution.

## Dive Curated Terms

The following phenotypic, genotypic, and functional terms are of significance to the work described in this paper:

FSD1 Gramene: At4g25100
FSD1 Araport: At4g25100

MSD1 Gramene: AT3G10920
MSD1 Araport: AT3G10920

FSD2 Gramene: AT5G51100
FSD2 Araport: AT5G51100

FSD3 Gramene: AT5G23310
FSD3 Araport: AT5G23310

CPN20 Gramene: AT5G20720
CPN20 Araport: AT5G20720

nudt7 Gramene: AT4G12720
nudt7 Araport: AT4G12720

CLSM Gramene: Confocal Laser-Scanning Microscopy
CLSM Araport: Confocal Laser-Scanning Microscopy
