# Supplementary material for: RACK1A interacts and colocalizes with FSD1 in stress granules to regulate salt stress response in Arabidopsis
Source: Plant Physiol. 2025 Dec 17;200(1):kiaf659. doi: 10.1093/plphys/kiaf659 (PMC12853880; doi:10.1093/plphys/kiaf659)
Supplement: kiaf659_Supplementary_Data [file kiaf659_supplementary_data.zip › Melicher et al supplementary material.pdf]

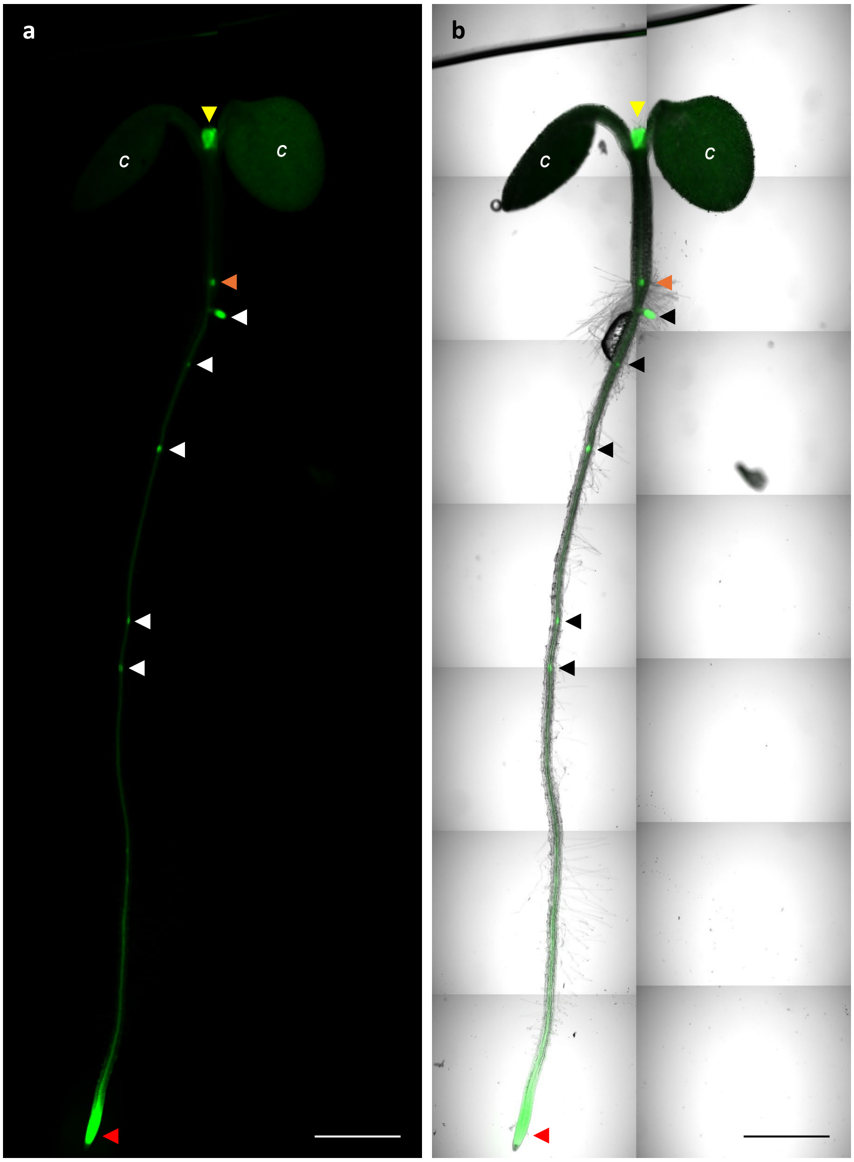

Supplementary Figure S1. Localization of RACK1A-GFP in 5-day-old seedling as observed using fluorescence ZOOM stereomicroscope. (a) RACK1A-GFP fluorescence detected in the GFP channel. **(b)** Merged images of detected GFP fluorescence and transmitted light. Both composite images are assembled from 14 separate images each, using a Panorama function of the ZEN 3.4 software (Carl Zeiss, Germany). c – cotyledons, yellow arrowheads indicate true leaves, orange arrowheads indicate forming adventitious root, white a) or black (b) arrows indicate lateral root primordia and growing lateral roots, red arrowheads indicate root tip. Scale bar = 1000 µm.

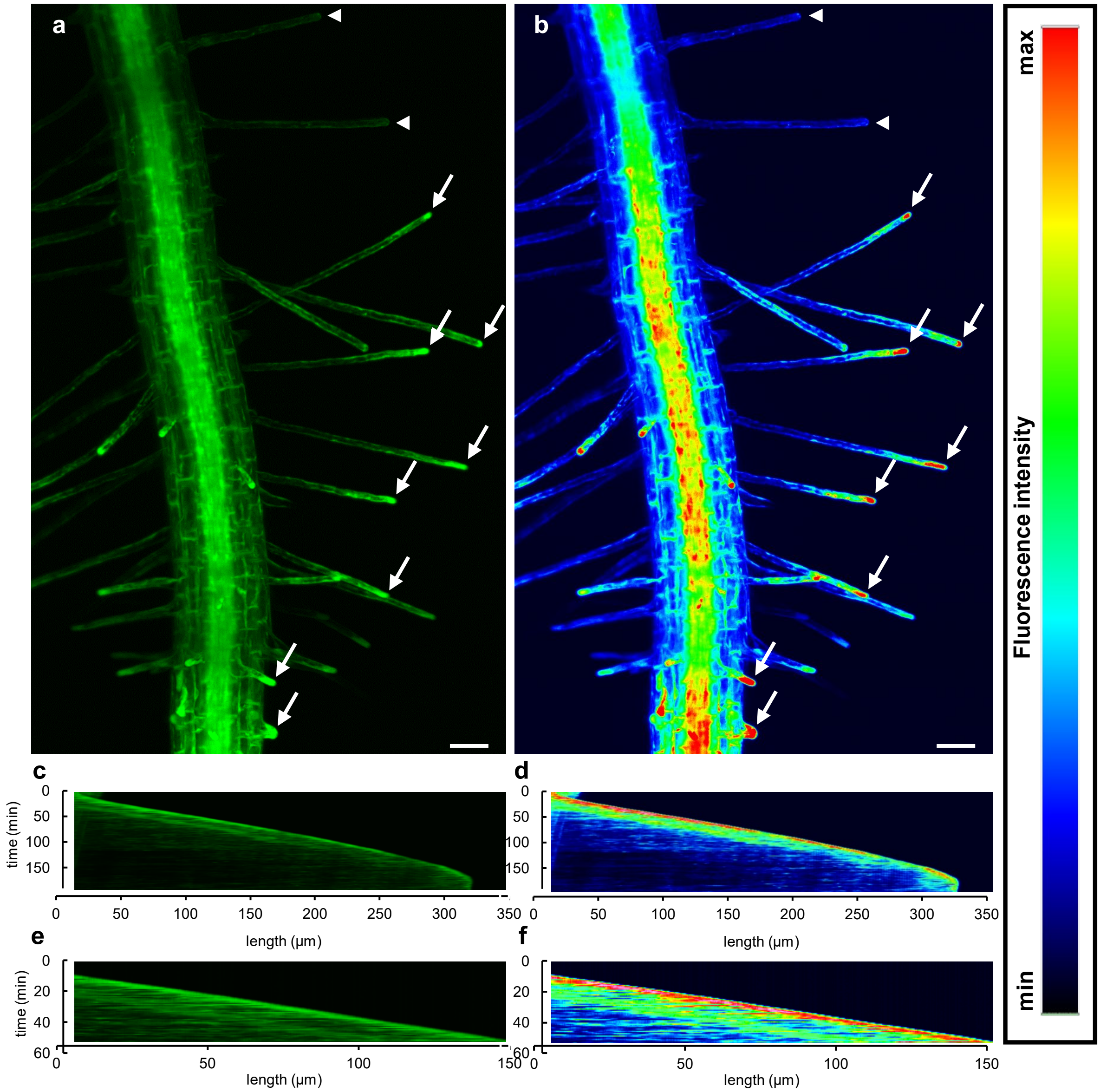

Supplementary Figure S2. Microscopic observation of RACK1A-GFP subcellular localization during root hair development and tip growth. (a, b) *In vivo* dynamic localization of RACK1A-GFP in growing primary root visualized using light-sheet fluorescence microscopy in a root hair-formation zone. RACK1A-GFP (a) and pseudocolor (b) fluorescence intensity distribution at different stages of root hair development showed a tip-focused localization of RACK1A-GFP in bulges, emerging, and actively growing root hairs (arrows), while it disappeared from the tip in fully-grown root hairs with terminated tip growth (arrowheads). **(c-f)** Quantitative representation of root hair tip growth rate in kymographs of selected root hair until the termination of its tip growth (c, d), and depicted phase of its fast growth (e, f). Tip-focused fluorescence signal is presented as either RACK1A-GFP (c, e) or pseudocolor (d, f) fluorescence intensity distribution profile. Kymographs present measurements in 187 min and 350 µm (c, d), and 60 min and 150 µm (e, f) intervals. Heat map shows RACK1A-GFP fluorescence intensity in pseudocolors with the lowest fluorescence intensity corresponding to the dark blue and the highest fluorescence intensity corresponding to the red color. Scale bar = 50 µm (a, b).

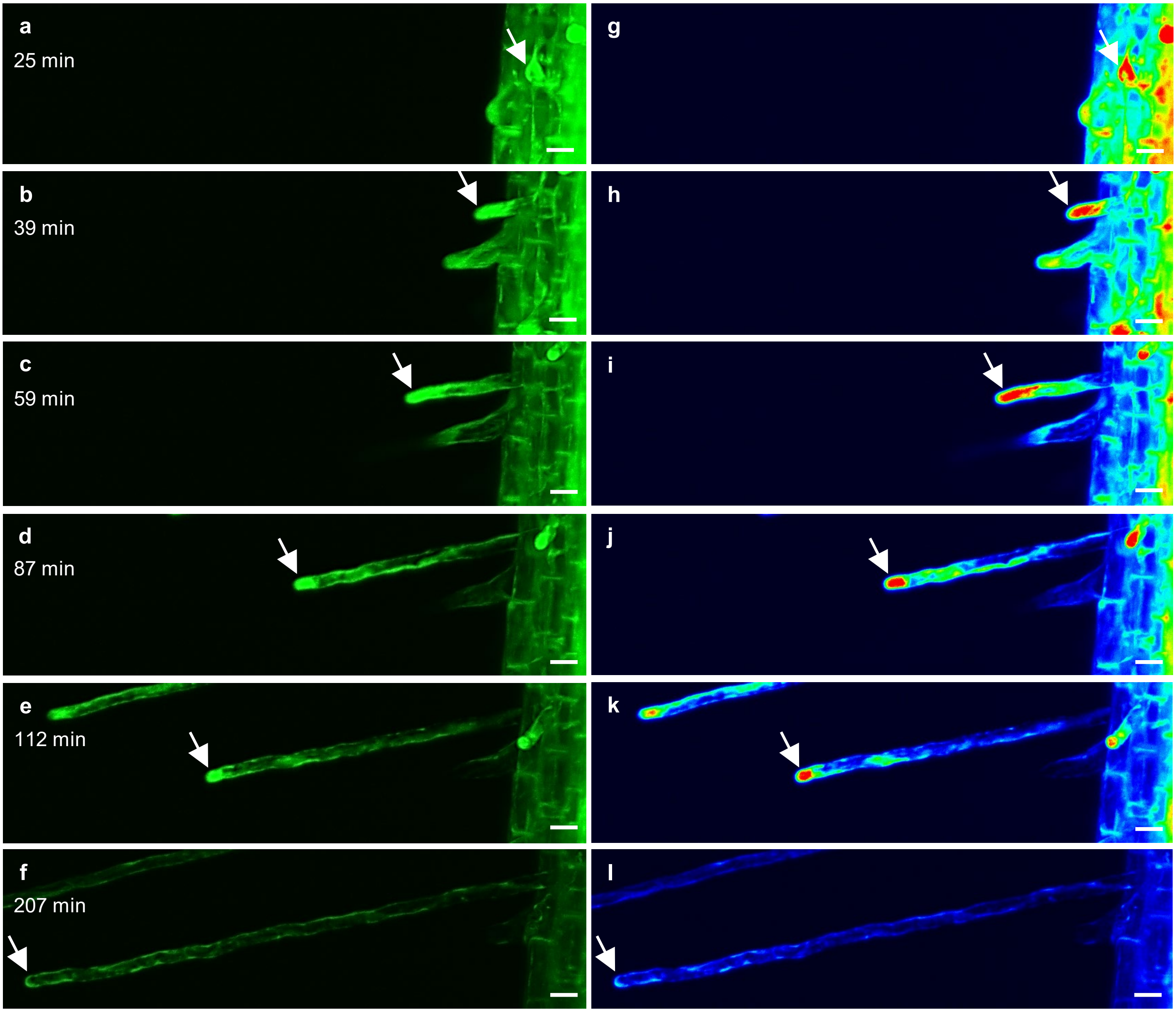

Supplementary Figure S3. Time-course imaging of RACK1A-GFP localization and accumulation during root hair formation. (a-f) Time-lapse recording of root hair development showing localization pattern of RACK1A-GFP and **(g-l)** a semi-quantitative signal intensity visualization by pseudo-color-coded range in different developmental stages including (a, g) bulge, actively growing (b, h) short and (c-e; i-k) longer root hairs, and (f, l) root hair with terminated tip growth. The pseudo-color-coded scale ranges from black representing minimal signal intensity to red representing maximum signal intensity. Time of root hair sequential imaging over the time period of 207 minutes is shown (in min) using light-sheet fluorescence microscope. Arrows point at RACK1A-GFP accumulation in (a-e; g-k) apical and subapical part of emerging and growing root hairs which is clearly absent in (f, l) non-growing root hair. Scale bar = 20 µm (a–l).

a

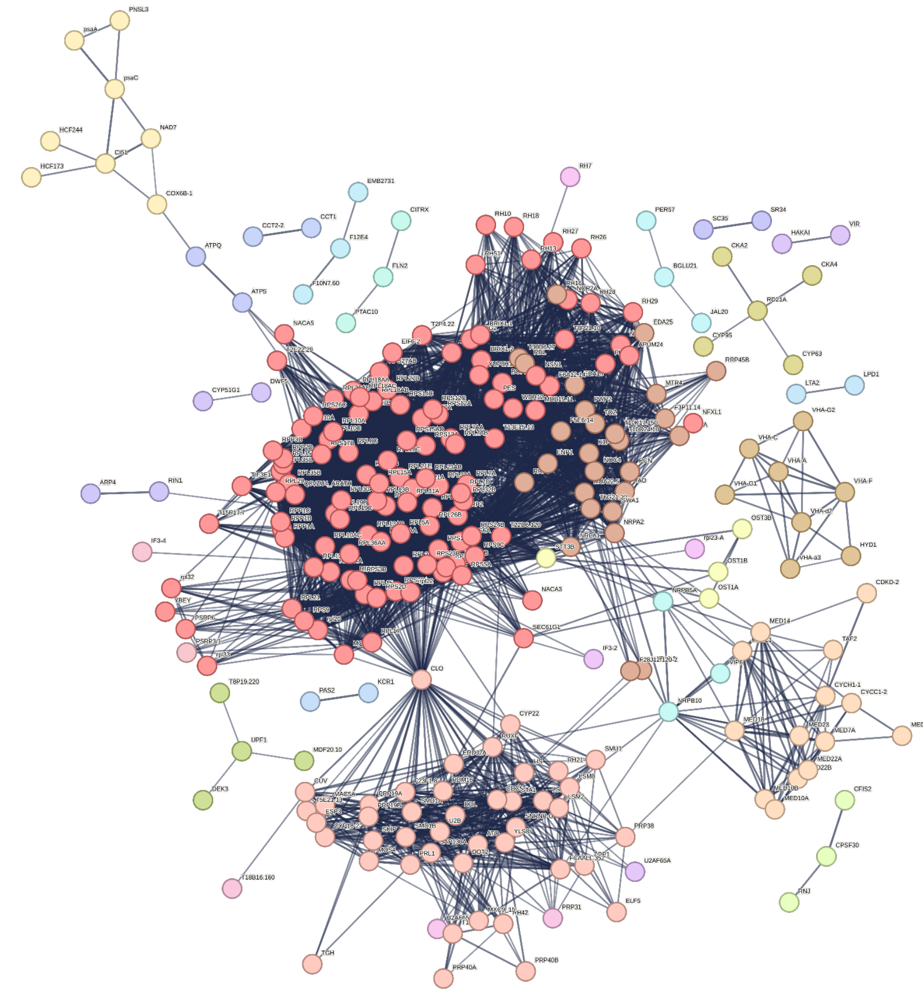

b

color	cluster Id	gene count	description
	Cluster 1	117	+ Ribosome
	Cluster 2	43	+ Spliceosome
	Cluster 3	27	+ Ribosome biogenesis
	Cluster 4	14	Mediator complex
	Cluster 5	8	+ Transferrin endocytosis and recycling
	Cluster 6	8	Photosystem
	Cluster 7	5	+ Circadian rhythm - plant
	Cluster 8	4	+ Various types of N-glycan biosynthesis
	Cluster 9	4	+ Regulation of double-strand break repair
	Cluster 10	3	Processing of Intronless Pre-mRNAs, and mRNA cleavage and polyadenylation spe...
	Cluster 11	3	+ Chloroplast nucleoid
	Cluster 12	3	+ RNA polymerase IV complex
	Cluster 13	3	BGLU21, JAL20, PER57
	Cluster 14	3	EMC complex

Supplementary Figure S4. Protein interaction networks **(a)** and the annotation of protein clusters **(b)** assigned to the interactome of RACK1A-GFP after mock treatment by STRING analysis. K-means clustering was applied to the network constructed from 536 proteins identified by co-immunoprecipitation analysis of RACK1A-GFP interactome. Experimentally proved interactions and co-expression were considered using a high confidence score (0.7) to evaluate protein interaction networks in STRING. Only clusters composed of min. 3 nodes are annotated.

a

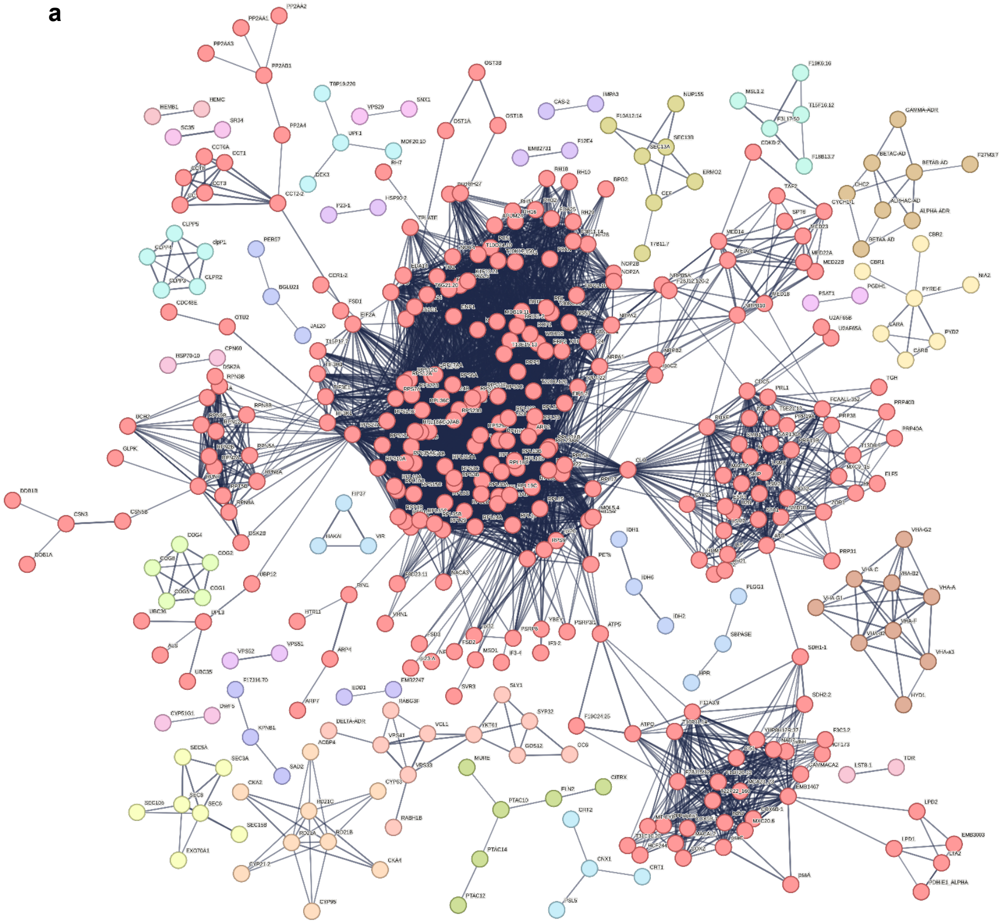

b

color	cluster Id	gene count	description
	Cluster 1	285	Ribosome
	Cluster 2	11	Vesicle fusion
	Cluster 3	9	+ Transferrin endocytosis and recycling
	Cluster 4	9	+ Mixed, incl. Peptidase C1A, papain C-terminal, and Receptor Mediated Mitophagy
	Cluster 5	8	+ AP-type membrane coat adaptor complex
	Cluster 6	7	+ Pyrimidine metabolism
	Cluster 7	7	COPII-coated vesicle budding
	Cluster 8	7	+ Exocytosis
	Cluster 9	6	+ Chloroplast nucleoid
	Cluster 10	5	Retrograde transport at the Trans-Golgi-Network
	Cluster 11	5	+ intra-Golgi vesicle-mediated transport
	Cluster 12	5	+ Protein quality control for misfolded or incompletely synthesized proteins
	Cluster 13	4	+ Regulation of double-strand break repair
	Cluster 14	4	Calnexin/calreticulin cycle
	Cluster 15	3	mRNA methylation
	Cluster 16	3	Photorespiration
	Cluster 17	3	+ Isocitrate metabolism
	Cluster 18	3	BGLU21, JAL20, PER57
	Cluster 19	3	+ Importin-beta N-terminal domain

Supplementary Figure S5. Protein interaction networks **(a)** and the annotation of protein clusters **(b)** assigned to the interactome of RACK1A-GFP after salt treatment by STRING analysis. K-means clustering was applied to the network constructed from 998 proteins identified by co-immunoprecipitation analysis of RACK1A-GFP interactome. Experimentally proved interactions and co-expression were considered using a high confidence score (0.7) to evaluate protein interaction networks in STRING. Only clusters composed of min. 3 nodes are annotated.

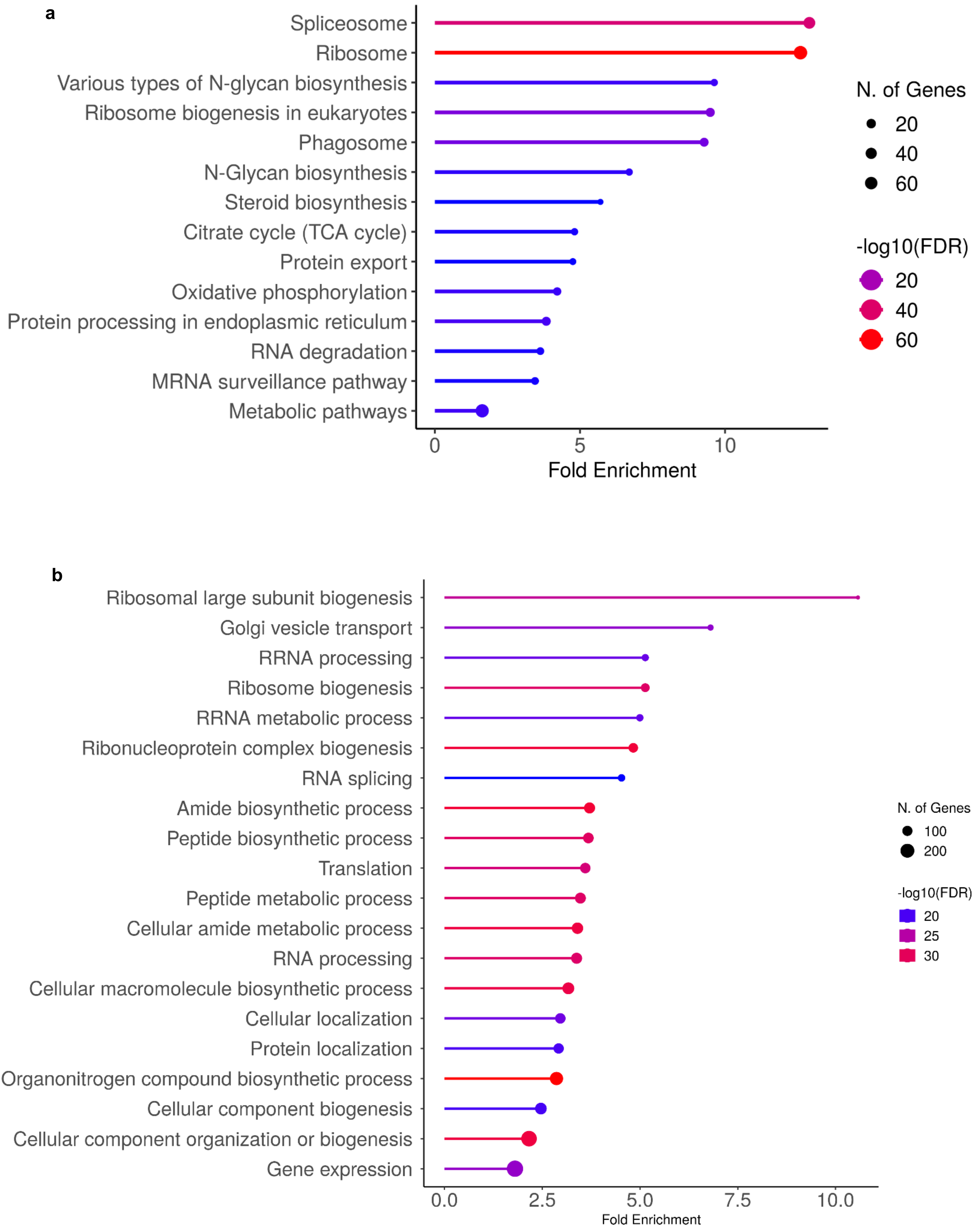

Supplementary Figure S6. Gene ontology annotation enrichment analysis of proteins identified by co-immunoprecipitation analysis of RACK1A-GFP in stably transformed *rack1a-1* mutants expressing *proRACK1A::RACK1A:GFP* construct, exposed to mock treatment **(a)** and 200 mM NaCl **(b)**.

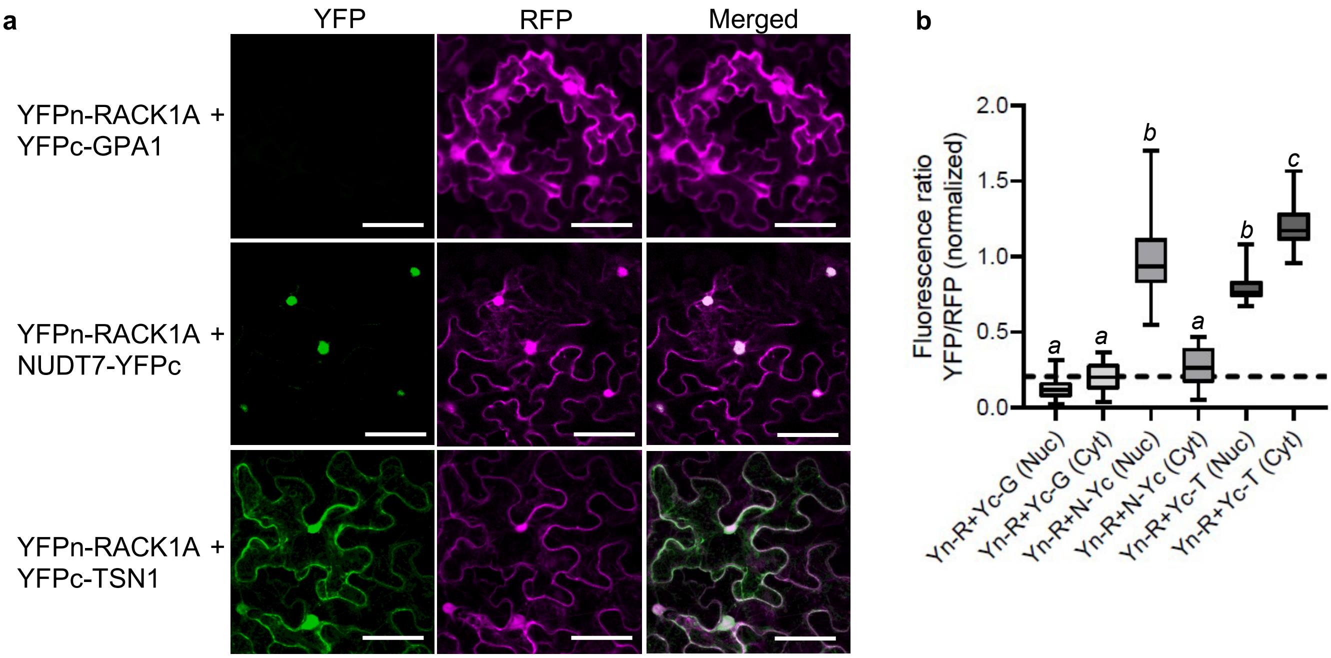

Supplementary Figure S7. Functional validation of RACK1A-TSN1 interaction. (a, b) Protein-protein interaction (PPI) study of RACK1A with TSN1 by ratiometric bimolecular fluorescence complementation assay (rBiFC). (a) Representative images of rBiFC assay taken by confocal laser scanning microscopy (CLSM). Artificial green color for YFP represents the PPI, RFP signal (magenta) functions as a control of transformation and reference for calculations of relative PPI strength. Scale bars = 20 µm. (b) Relative quantification of PPI strength measured as a ratio of YFP to RFP relative fluorescence intensity in nuclei (Nuc) and cytosol (Cyt). Ratio values were normalized to the mean ratio measured in nuclei of positive control transformed with vectors carrying *YFPn-RACK1A+NUDT7-YFPc* (Yn-R+N-Yc) genes. The dashed line represents the mean ratio measured in negative controls transformed with vectors carrying YFPn-RACK1A + YFPc-GPA1 (Yn-R+Yc-G) and functions as a threshold of positive interaction. Vector carrying *YFPn-RACK1A+TSN1-YFPc* (Yn-R+T-Yc) genes was used to study the interaction of RACK1A with TSN1. Centre lines of the boxes represent median values. The upper and lower box limits represent the upper and lower quartiles, respectively. Whiskers represent maximum and minimum values. Italic letters indicate a statistically significant difference at a $p < 0.05$ as determined by one-way ANOVA with *post hoc* Tukey HSD test.

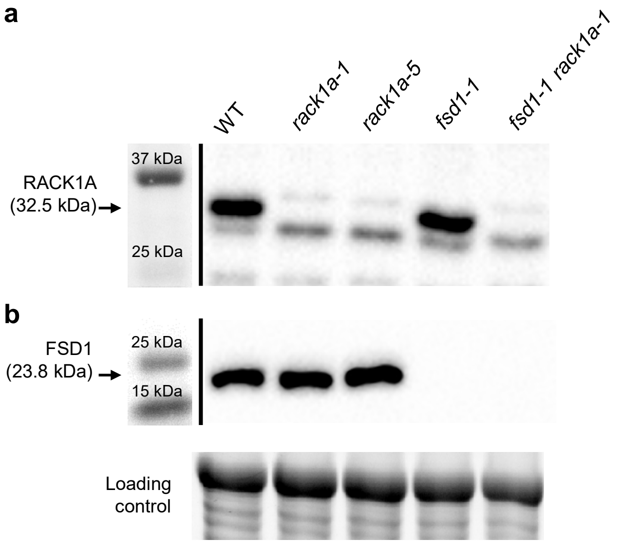

Supplementary Figure S8. Validation of *rack1a-5* and *fsd1-1 rack1a-1* mutants by immunoblotting. Immunoblots of RACK1A **(a)** and FSD1 **(b)** in *Arabidopsis* Col-0 (wild type; WT), *rack1a-1*, *rack1a-5, fsd1-1* and *fsd1-1 rack1a-1* are supplemented with control of protein loading as visualized on Stain-free gel, and protein standard lanes to estimate molecular weight of the proteins.

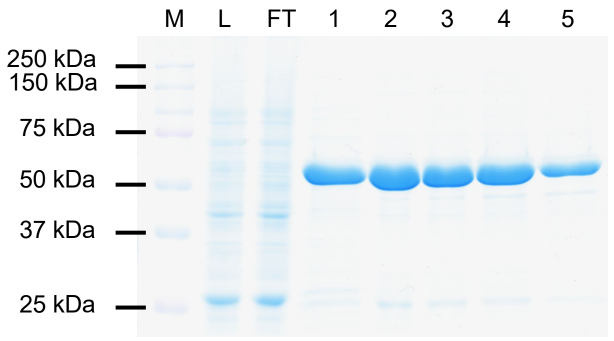

Supplementary Figure S9. Production, purification, and detection of recombinant GST-RACK1A. The production of recombinant RACK1A was performed using the *pGEX6P-1* vector in the bacterial strain *E. coli* BL21 Star (DE3). The produced recombinant GST-RACK1A protein was purified by affinity chromatography using Glutathione Sepharose 4B resin. Proteins from the obtained fractions, including bacterial lysate (L), flow-through (FT), and eluted fractions (1–5), were separated by SDS-PAGE and visualized with Coomassie staining. The relative mass of protein standards (Precision Plus ProteinTM Dual Color) are shown on the left (M) and the size of recombinant GST-RACK1A is approximately 60 kDa.

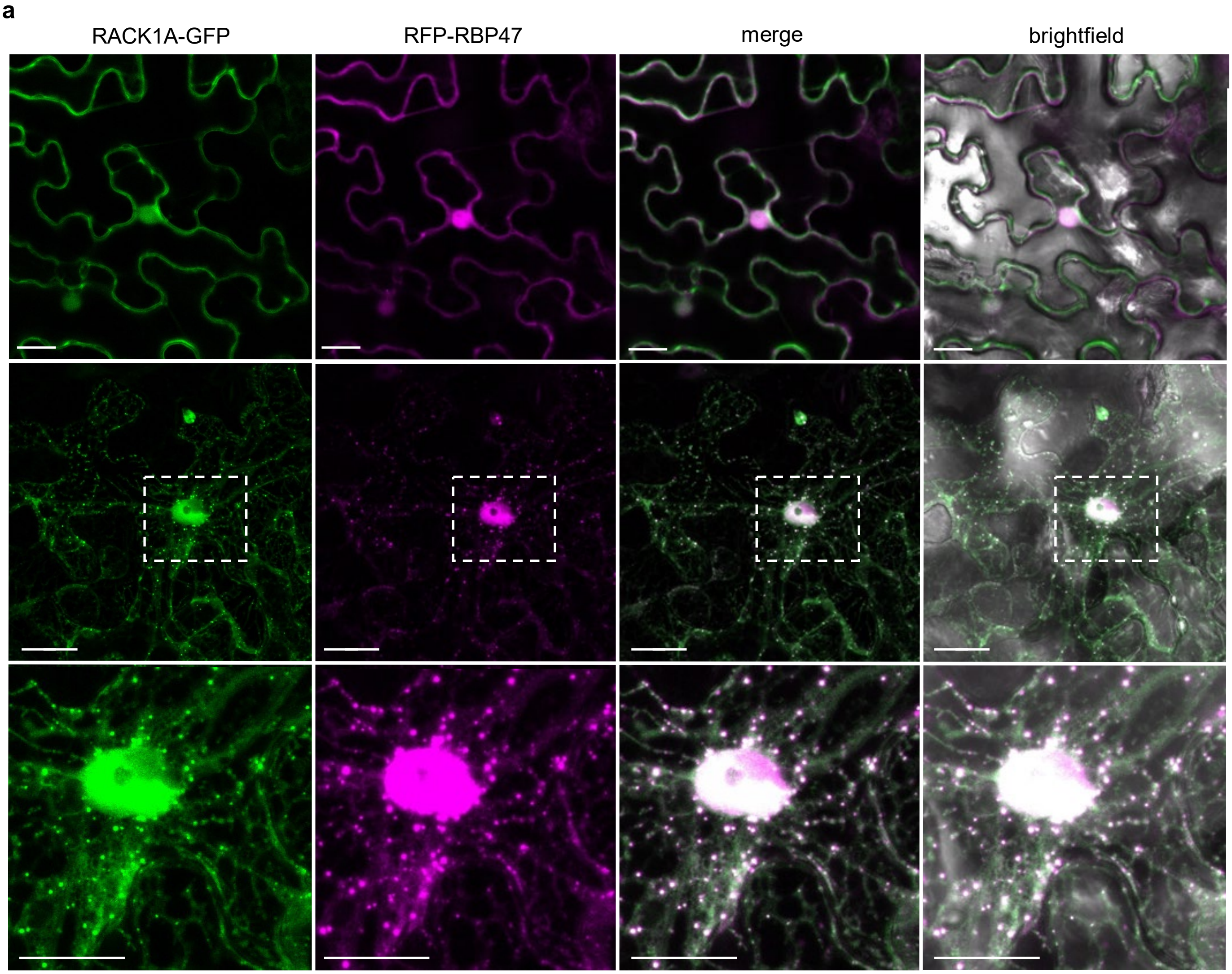

b

Pearson's correlation coefficient	The Manders' colocalization coefficient
0.92 ± 0.02	0.97 ± 0.01

Supplementary Figure S10. Salt stress induced colocalization of RACK1A-GFP and stress granule marker RFP-RBP47 in transiently transformed *N. benthamiana* epidermal leaf cells. (a) Representative images of epidermal leaf cells co-transformed with RACK1A-GFP (green) and RFP-RBP47 (magenta) constructs, treated with ½ MS medium or ½ MS medium supplemented with 200 mM NaCl for 30 min. The lower images are magnified regions of the salt-stressed leaf images, along with their magnified regions with an adjusted intensity of the fluorescent signal. **(b)** Averaged Pearson's and Manders' correlation coefficients from colocalization analysis of RACK1A-GFP and RFP-RBP47 during salt stress-induced formation of stress granules (N = 70). Scale bar = 20 µm.

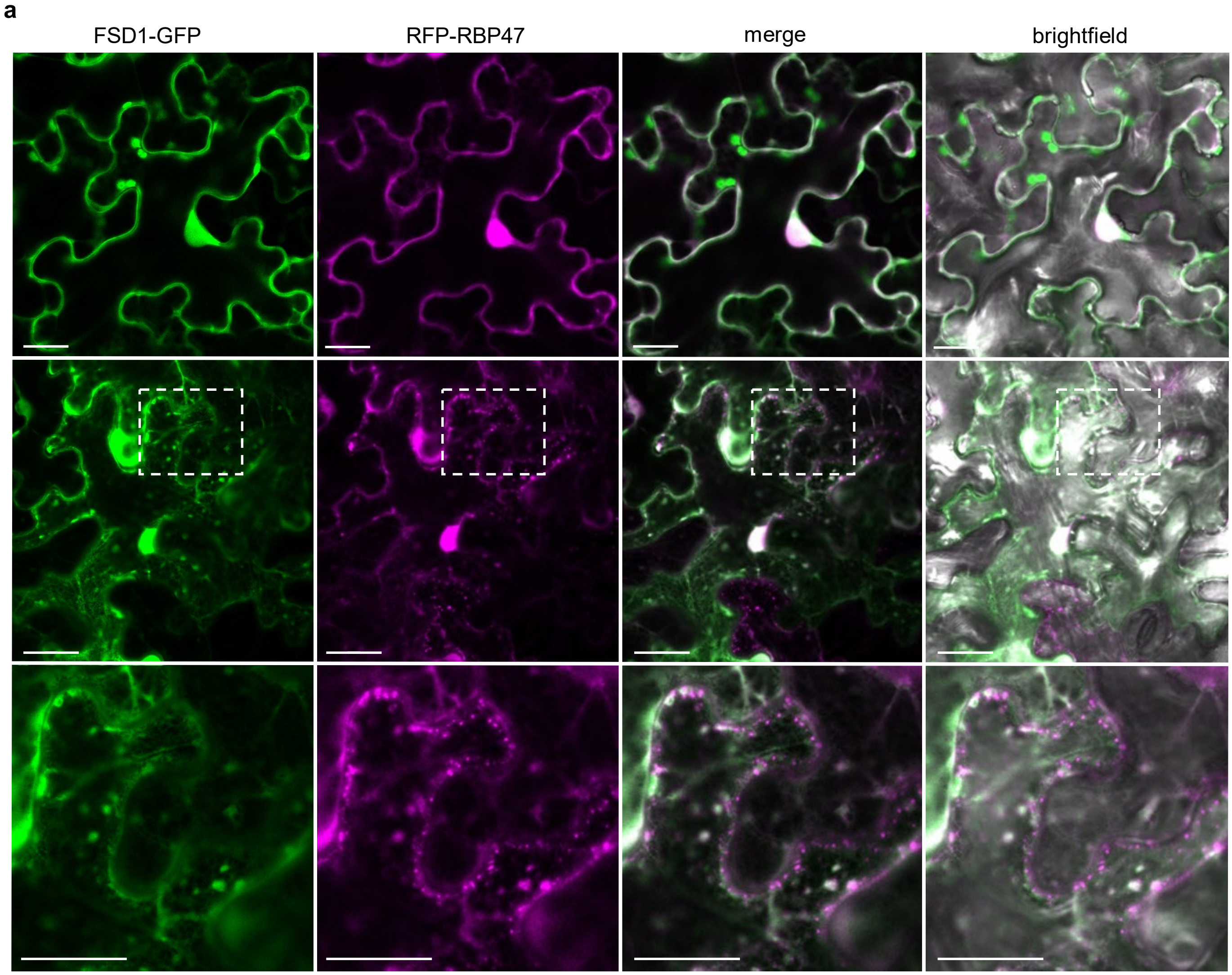

b

Pearson's correlation coefficient	The Manders' colocalization coefficients
0.87 ± 0.03	0.96 ± 0.01

Supplementary Figure S11. Salt stress induced colocalization of FSD1-GFP and stress granule marker RFP-RBP47 in transiently transformed *N. benthamiana* epidermal leaf cells. (a) Representative images of epidermal leaf cells co-transformed with FSD1-GFP (green) and RFP-RBP47 (magenta) constructs, treated with ½ MS medium or ½ MS medium supplemented with 200 mM NaCl for 30 min. The lower images are magnified regions of the salt-stressed leaf images, along with their magnified regions with an adjusted intensity of the fluorescent signal. **(b)** Averaged Pearson's and Manders' correlation coefficients from colocalization analysis of FSD1-GFP and RFP-RBP47 during salt stress-induced formation of stress granules (N = 70). Scale bar = 20 µm.

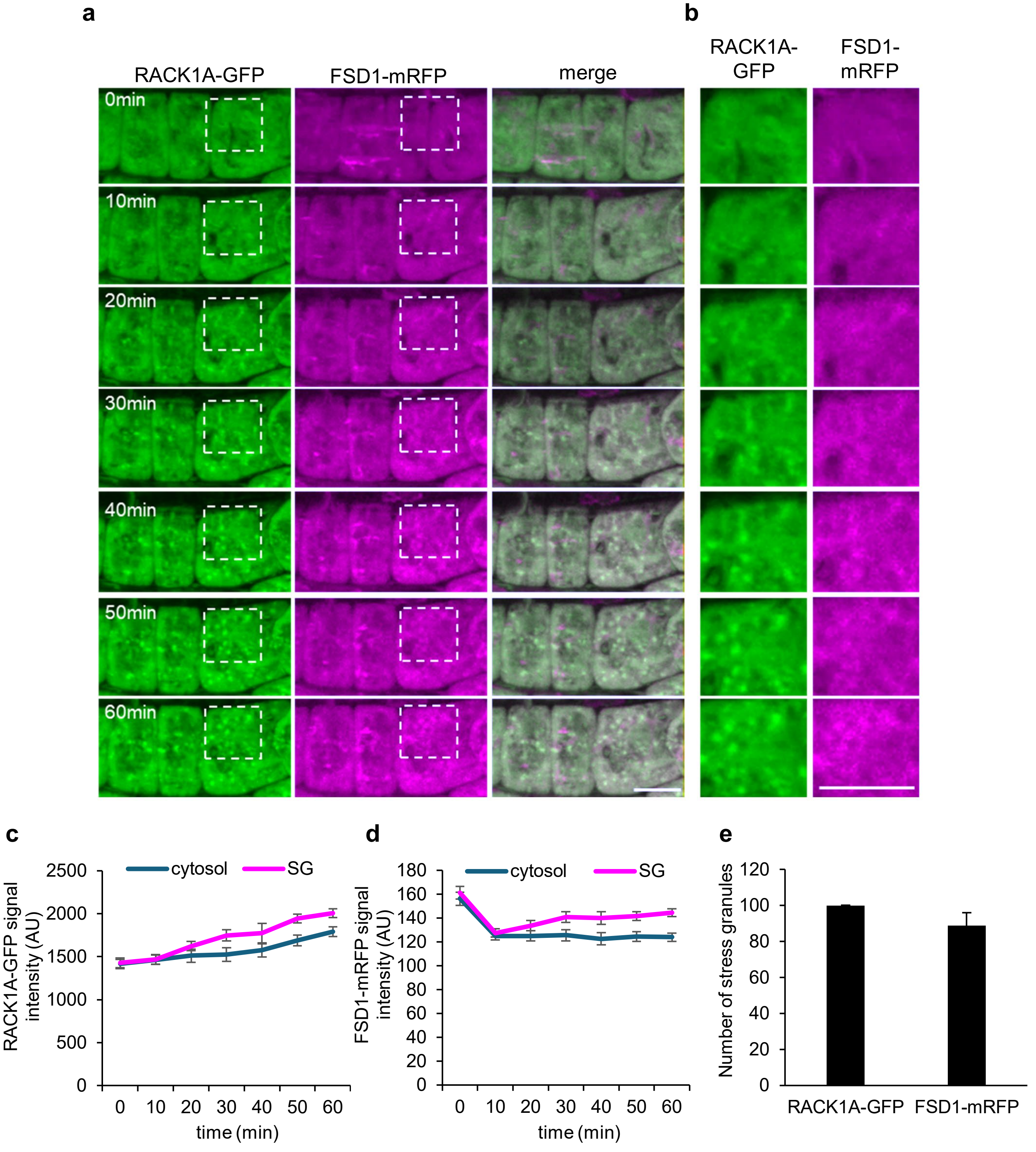

Supplementary Figure S12. Time course monitoring of NaCl-dependent changes in RACK1A-GFP and FSD1-mRFP fluorescence in *rack1a* mutant expressing *proRACK1A::RACK1A:GFP* and *proFSD1::FSD1:mRFP* constructs. (a, b) Overview (a) and detailed view (b) showing areas highlighted by boxes in A of cortical cytoplasm in epidermal cells of the root meristematic and transition and meristematic zones of the genetically-rescued *rack1a-1* mutant showing distribution of RACK1A-GFP (green), FSD1-mRFP (magenta) and their overlay (merge). Images were acquired every 10 min up to 60 min from samples treated with ½ MS medium supplemented with 100 mM NaCl. Scale bar = 10 µm. **(c, d)** Quantification of cytosolic and stress granule (SG)-specific fluorescence signal intensity, specific for RACK1A-GFP (c) and FSD1-mRFP (d) in A. The signal was measured using a „Measure“ function of the ZEN3.12 software in at least 10 regions of interest per cell in cytosol and at least 10 stress granules per cell. Five cells were quantified per seedling, in 3 seedlings in total. **(e)** Relative quantification of SG number containing red and green signals (5 cells in 3 plants were included in quantification). Error bars in C-E represent standard deviation.
