# Supplementary material for: RACK1A interacts and colocalizes with FSD1 in stress granules to regulate salt stress response in Arabidopsis
Source: Plant Physiol. 2025 Dec 17;200(1):kiaf659. doi: 10.1093/plphys/kiaf659 (PMC12853880; doi:10.1093/plphys/kiaf659)
Supplement: kiaf659_Supplementary_Data [file kiaf659_supplementary_data.zip › PLPHYS-2025-1153R2_Supplementary video titles.pdf]

Supplementary Video S1. Time-lapse imaging of primary root growth of RACK1A-GFP lines using LSFM.

Supplementary Video S2. Time-lapse imaging of developing root hairs in the root of RACK1A-GFP line using LSFM.

Supplementary Video S3. Time-lapse imaging of primary root growth of RACK1A-GFP lines using LSFM in a pseudo-color-coded semi-quantitative fluorescence intensity distribution.

Supplementary Video S4. Time-lapse imaging of developing root hairs in the root of RACK1A-GFP line using LSFM in a pseudo-color-coded semi-quantitative fluorescence intensity distribution.

Supplementary Video S5. Time-lapse imaging of developing lateral root primordia in the root of RACK1A-GFP line using LSFM.

Supplementary Video S6. Time-lapse imaging of developing lateral root primordia in the root of RACK1A-GFP line using LSFM in a pseudo-color-coded semi-quantitative fluorescence intensity distribution.
